# Body Size Poorly Predicts Host-Associated Microbial Diversity in Wild Birds

**DOI:** 10.1128/spectrum.03749-22

**Published:** 2023-04-11

**Authors:** Elizabeth A. Herder, Heather R. Skeen, Holly L. Lutz, Sarah M. Hird

**Affiliations:** a Department of Molecular and Cell Biology, University of Connecticut, Storrs, Connecticut, USA; b Department of Ecology and Evolutionary Biology, University of Connecticut, Storrs, Connecticut, USA; c Negaunee Integrative Research Center, Field Museum of Natural History, Chicago, Illinois, USA; d Department of Pediatrics, UC San Diego School of Medicine, La Jolla, California, USA; e Institute for Systems Genomics, University of Connecticut, Storrs, Connecticut, USA; Wayne State University

**Keywords:** microbiome, microbiota, island biography, species-area relationship, avian, bird, avian microbiome

## Abstract

The composition and diversity of avian microbiota are shaped by many factors, including host ecologies and environmental variables. In this study, we examine microbial diversity across 214 bird species sampled in Malawi at five major body sites: blood, buccal cavity, gizzard, intestinal tract, and cloaca. Microbial community dissimilarity differed significantly across body sites. Ecological theory predicts that as area increases, so does diversity. We tested the hypothesis that avian microbiota diversity is correlated with body size, used as a proxy for area, using comparative phylogenetic methods. Using Pagel’s lambda, we found that few microbial diversity metrics had significant phylogenetic signals. Phylogenetic generalized least squares identified a significant but weak negative correlation between host size and microbial diversity of the blood and a similarly significant but weakly positive correlation between the cloacal microbiota and host size among birds within the order *Passeriformes*. Phylosymbiosis, or a congruent branching pattern between host phylogeny and their associated microbiota similarity, was tested and found to be weak or not significant in four of the body sites with sufficient sample size (blood, buccal, cloaca, and intestines). Taken together, these results suggest that the avian microbiome is highly variable, with microbiota diversity demonstrating few clear associations with bird size. Finally, the blood microbiota have a unique relationship with host size.

**IMPORTANCE** All animals coexist and interact with microorganisms, including bacteria, archaea, microscopic eukaryotes, and viruses. These microorganisms can have an enormous influence on the biology and health of macro-organisms. However, the general rules that govern these host-associated microbial communities are poorly described, especially in wild animals. In this paper, we investigate the microbial communities of over 200 species of birds from Malawi and characterize five body site bacterial microbiota in depth. Because the evolutionary relationships of the host underlie the relationship between any host-associated microbiota relationships, we use phylogenetic comparative methods to account for this relationship. We find that the size of a host (the bird) and the diversity and composition of the microbiota are largely uncorrelated. We also find that the general pattern of similarity between host phylogeny and microbiota similarity is weak. Together, we see that bird microbiota are not strongly tied to host size or evolutionary history.

## INTRODUCTION

A microbiome is both a trait of the host (1) and an ecological community comprised of microorganisms capable of complex and dynamic interactions (2). Microbiota influence and are influenced by their host’s biology in numerous ways and vertebrates are a prominent global habitat for microbes. Identifying how micro- and macro-organisms coexist is fundamental to understanding biodiversity. Yet, drivers of community organization of host-associated microbiota in wild animals are virtually unknown, including how well these microbial communities follow established ecological theory.

Ecological theories provide specific hypotheses for testing and inferring rules of life (3), including ecological processes that are essential to understanding the composition, resilience, and evolution of the microbiome (4). A general theory of the species-area relationship, where larger areas tend to contain more biodiversity than smaller areas, is one of the best-supported “laws” in ecology (5, 6). Complementary to this law is a generally predictable pattern of biodiversity accumulation, which has been generalized to a diversity-area relationship (7). Whether microbiota, which are constantly under surveillance and management by host immune systems, adhere to the diversity-area relationship is an open question. Several studies have demonstrated that body size positively correlates with microbiota richness and diversity (8, 9), implying adherence of microbiota to diversity-area relationships. However, the nonindependence of host species was not accounted for in these cases. Because the microbiome is a trait of its host as well as an ecological community (1), we must account for the underlying phylogeny of the hosts to understand trait evolution (10). To appropriately test the diversity-area relationship between host size (a proxy for area) and microbial biodiversity, as well as to ensure any correlations are not simply a factor of the relatedness of host species, phylogenetic comparative analyses are used. Assessing the results from standard and phylogenetic comparative methods bears on whether the framework of “the microbiota are a trait of the host” is warranted.

Birds (class: *Aves*) are important members of Earth’s biosphere and bird body size spans five orders of magnitude by weight, making them an excellent clade for exploring diversity-area relationships in host-associated microbiota. Birds have diverse ecologies which affect their microbiota (11). Similarly, morphological adaptations corresponding to specific diets and behaviors impact microbiota composition and diversity (e.g., reference 12). Microbes can also provide protection against invading pathogens, facilitate nutrient uptake, or influence birds’ behavior (13, 14). A holistic understanding of avian biology requires knowledge of their microbes, both across bird species and across anatomical body sites.

**FIG 1:**
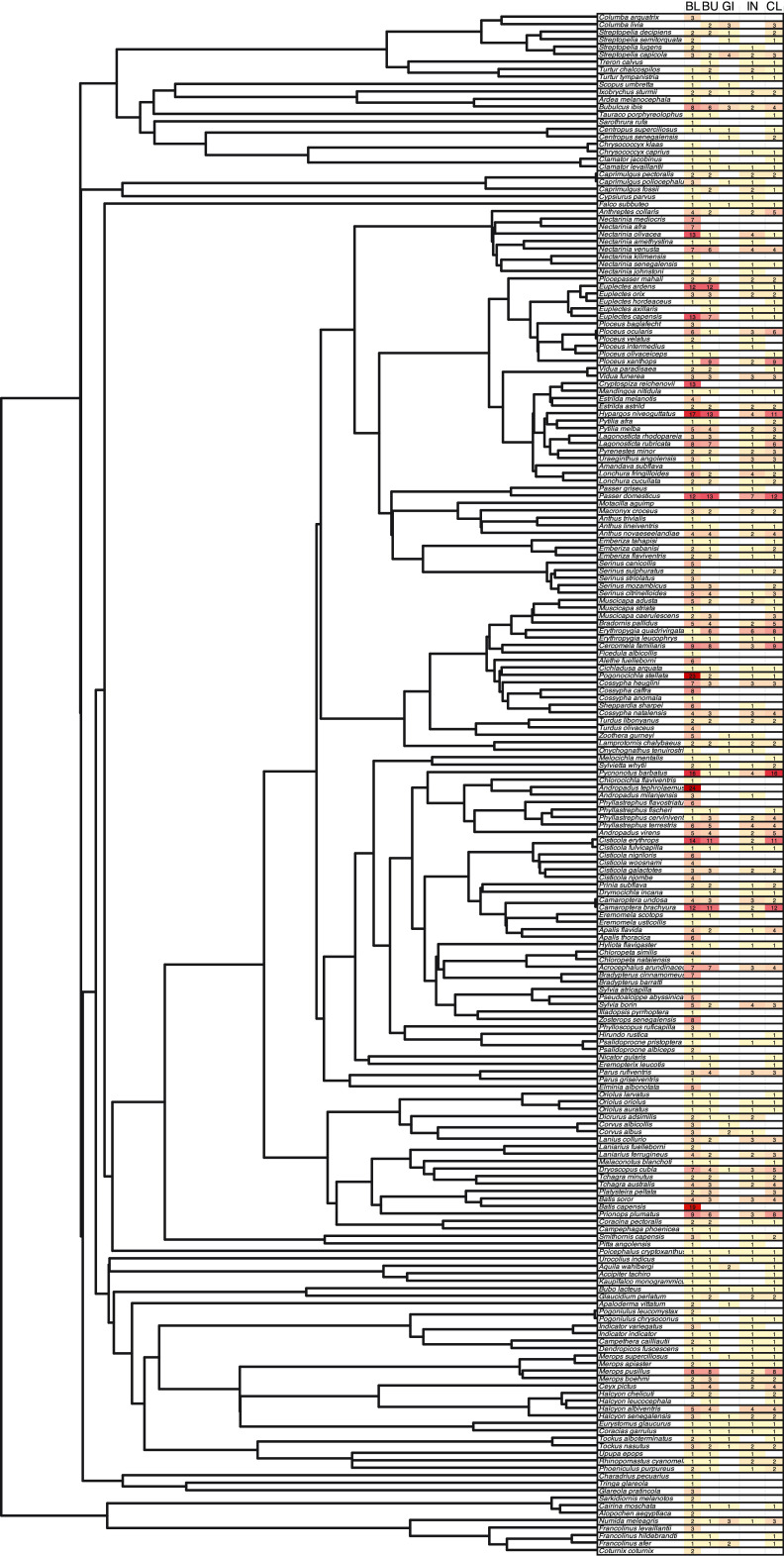
Phylogeny of the bird species represented in this study (left) and a table with sample counts for each sample (right). Darker red represent higher sample sizes; lighter yellow are lower. Data are in table format in Table S2.

In this study, we characterize the microbiota of five body sites from diverse avian hosts (Fig. 1; Table S1 in the supplemental material). Four of these are distinct sites along the gastrointestinal tract, connected to each other through digestion: buccal (oral cavity), gizzard, intestines, and cloaca. The gastrointestinal tract is frequently exposed to external microbes through the intake and digestion of food materials. Despite all sections of the gastrointestinal tracts sharing input, each region harbors unique microbial communities (15). These body sites may follow diversity-area relationships to various degrees and may uniquely reflect host taxonomy. The remaining body site in our study, blood, is more rarely in contact with novel microbes than the gastrointestinal sites and thus will likely have fewer immigrants in its microbiota.

In addition to a broad description of bird-associated microbiota, there were several specific goals of this study. First, we compare the taxonomic composition and diversity of the microbiota of hundreds of birds across multiple body sites for the purpose of documenting microbiodiversity. This includes statistical assessment of microbial alpha diversity, beta diversity, and the relationship between the microbiota and taxonomic, ecological, and individual host traits. Second, we quantify phylosymbiosis, the extent to which the similarity of the microbiota reflects that of the phylogeny of the host. Third, we estimated the phylogenetic signal of microbiota diversity using Pagel’s lambda. Fourth, we assessed the relationship between host size and microbiota diversity, using a phylogenetically controlled method to test the ecological theory relating to Diversity-Area and species-area relationships.

We had several nonexclusive expectations for these data. First, we expected a positive relationship between bird body size and microbiota diversity at all body sites, based on the Species-Area and diversity-area relationships. For these analyses, we assumed a general correlation between organ size and bird mass. While there are certainly exceptions, this assumption has worked well in previous comparative analyses of the gut, as the general size of the gut scales approximately with host size (9). Second, we expected there to be a stronger (i.e., more statistically significant) positive relationship between bird size and microbiota at the gastrointestinal sites than in blood, due to the shared immune functions protecting blood from pathogens. An alternative expectation was that higher immune cell output of larger birds (16) may inhibit the establishment of new microbial colonizers and lead to lower than expected microbial diversity or richness, in which case neither blood nor gastrointestinal sites microbiota would show a positive correlation with host size.

## RESULTS

### Sequencing and taxonomy.

**FIG 2:**
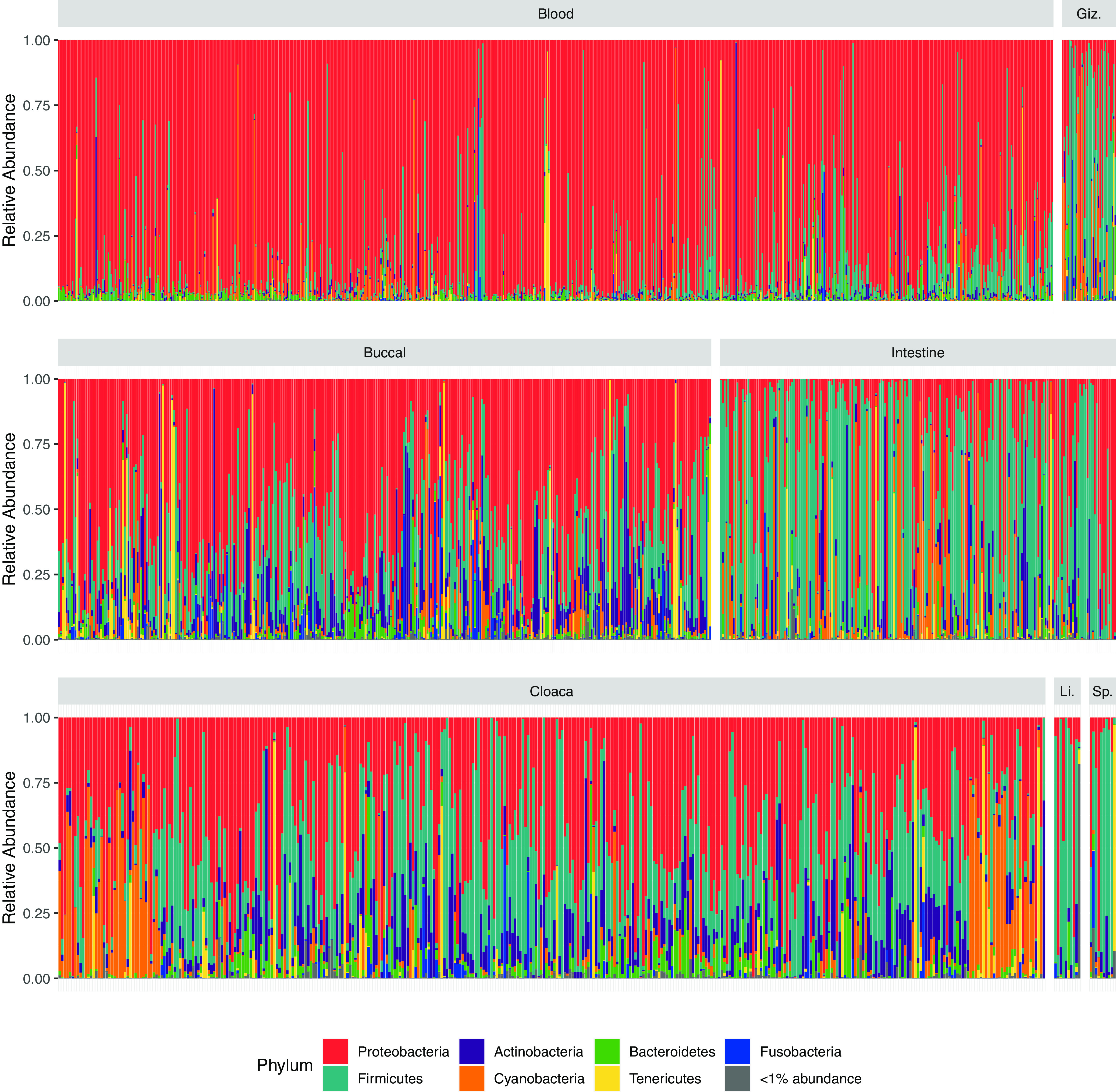
Phylum-level relative abundance stacked bar charts of all samples rarefied to 4,000 reads and grouped by body sites (Giz., Gizzard; Li., Liver; and Sp., Spleen) as a broad scale overview of the microbial diversity within specific body sites. Phyla with total abundance less than 1% are summed together and represented by the gray bar. Relative abundance bar charts of body site sorted by host taxonomic order and family are show in Fig. S2. Precise individual level data are in Table S3.

A total of 1,740 microbial 16S rRNA amplicon libraries were processed and analyzed from 214 bird species, 756 individual birds, and a total of 7 body sites, including blood (*n* = 723), buccal (*n* = 358), cloaca (*n* = 377), gizzard (*n* = 45), intestines (*n* = 217), liver (*n* = 10), and spleen (*n* = 10). In total, the full data set included 87,239,296 reads with an average read depth of 65,595 reads (SD ± 47,441 reads). Individual samples contained an average of 115.8 amplicon sequence variants (ASVs) (SD ± 123.8). The full data set included 34,944 ASVs from 46 phyla, 129 classes, 231 orders, 328 families, and 688 genera. Taxonomic composition across all body sites was dominated by *Proteobacteria* and *Firmicutes*, although the relative abundance of these and other phyla differed between body sites (Fig. 2) (Fig. S2 and Table S3).

### Alpha diversity.

**FIG 3:**
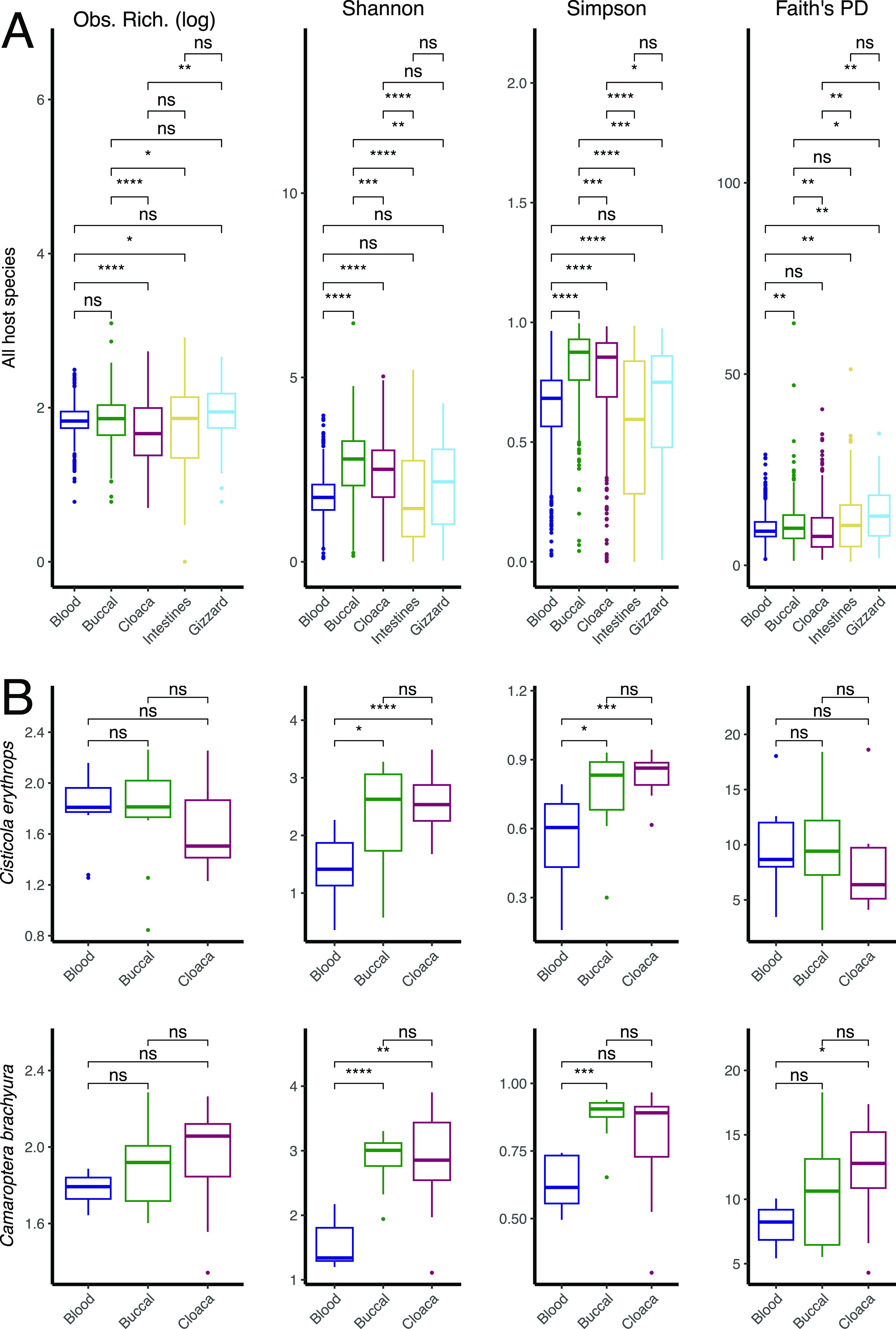
(A) Box plots of alpha diversity of all samples, separated by sample type (body site) for all four metrics observed ASVs, Shannon, Simpson, and Faith’s PD. (B) Alpha diversity of two species, *Cisticola erythrops* (*n* = 14 blood, 11 buccal, 11 cloaca) and *Camaroptera brachyura* (*n* = 12 blood, 11 buccal, and 12 cloaca). Significance levels are pairwise comparisons between body sites: ns, *P* > 0. 05; *, *P* < 0.05; **, *P* < 0.01; ***, *P* < 0.001; ****, *P* < 0.0001.

Blood, buccal, cloaca, gizzard, and intestine samples were used to investigate variations in microbial diversity and assess whether, at the scale of class *Aves*, body sites are significantly similar in diversity and community composition. Samples were rarefied to 4,000 sequences (based on rarefaction curves, Fig. S1), and libraries that fell below 4,000 reads were excluded, resulting in the removal of 117 blood, 5 buccal, 8 cloaca, and 4 intestine samples. The final data set of rarefied libraries includes 606 blood samples, 353 buccal samples, 369 cloaca samples, 213 intestine samples, and 45 gizzard samples. The four measures of alpha diversity (observed ASV richness, Shannon diversity index, Simpson index, and Faith’s phylogenetic diversity [PD]) were generally variable across body sites (Fig. 3A) and across species (Fig. S3; Fig. S4). No specific body site was consistently the most or least diverse. Of all pairwise comparisons between body sites, only two comparisons were consistent across all four measures of diversity; intestines and gizzard microbiota were never significantly different while buccal and cloaca microbiota were always significantly different. The intraspecific variation of three body sites (blood, buccal, and cloaca) was also highly variable, with no consistent patterns (e.g., significant differences between alpha diversity of body sites) observed across host species (Fig. 3B; Fig. S4).

### Beta diversity.

**FIG 4:**
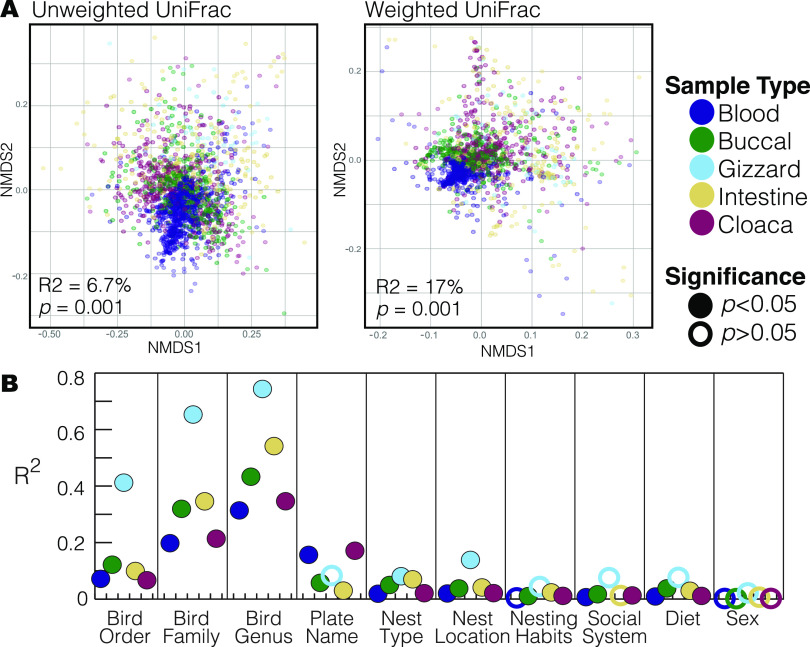
(A) Nonmetric Multidimensional Scaling ordination constructed from unweighted UniFrac and weighted UniFrac matrices of wild bird microbiomes collected from Malawi. Colors represent the five body sites sampled; results from the adonis tests for significance of body sites are shown. (B) Effect sizes of various metadata variables on the microbiota (results are shown for weighted Unifrac distance; results using unweighted UniFrac were similar and full results are in Table S4). Notably, there were significant differences in group dispersions in most cases where *P < *0.05 (see Table S4).

Body site significantly explained variation of the microbiota for both weighted (*P* = 0.001, *R*^2^ =17.0%) and unweighted UniFrac (*P* = 0.001, *R*^2^ = 6.7%, Fig. 4A). Within body sites, we assessed the correlation between the microbiota and host taxonomy, ecology, behaviors, and sex (Fig. 4B; Table S4). The results of the majority of the permutational multivariate analysis of variance (PERMANOVA) analyses indicate significant variation within categories; however, in most cases, the dispersions of the two groups were also significantly different. Therefore, results must be interpreted cautiously as the significant variation observed may be due to the dispersion of samples or may be a real indicator of microbial variation. There were few overarching patterns in the beta-diversity analyses, with the exception of host sex, which was never significantly different (for all body sites and both diversity metrics). Host taxonomic levels (order, family, and genus) indicated significant differences in community composition and explained the most variation observed, with *R*^2^ ranging from 31.3% to 74.4% in weighted UniFrac and from 25.3% to 73.6% in unweighted UniFrac, depending on body site. Bird ecological and behavioral traits, including diet and nesting habitats, were inconsistently significant, depending on body site and diversity metric, for both PERMANOVA analysis and beta-dispersion comparison. These traits generally had low effect sizes, rarely explaining more than 5% of the variation observed for both weighted and unweighted UniFrac distances. The extraction plate explained a significant amount of variation within sample types, with the effect size varying from 3.0% to 17.1% for weighted and 3.8% to 11.9% for unweighted UniFrac distances.

### Differential abundance.

To assess the differential abundance of microbial taxa between body sites, we conducted a pairwise comparison of individual body sites, rarefying to the lower value in each pairwise comparison. Overall, 56 genera contained ASVs that were differentially abundant in various body sites (Table 1; Fig. S5). *Hydrogenophaga*, *Hyphomicrobium*, Mycobacterium, *Phenylobacterium*, and *Sediminibacterium* all contained ASVs that were significantly overabundant in all pairwise comparisons to the blood and in no other body sites. *Gallibacterium* and *Hylemonella* contained ASVs that were significantly overabundant in all pairwise comparisons to the buccal samples and in no other body sites. *Lactobacillus* contained ASVs that were significantly overabundant in all pairwise comparisons to the intestine and in no other body sites. The gizzard contained the fewest significant taxa. Many genera appeared significantly differentially abundant in multiple comparisons (e.g., Acinetobacter contained ASVs that were overabundant in blood, buccal, and cloaca samples).

**TABLE 1:** The 56 genera that contained differentially abundant ASVs in pairwise comparisons between the body sites^*a*^

Genus	Blood	Buccal	Gizzard	Intestine	Cloaca	Relevant studies
Acinetobacter	4	4			3	
*Methylobacterium*	4	4		1	1	20
*Stenotrophomonas*	4	4			2	
*Sphingobium*	4	4			1	20
Pseudomonas	4	4				20, 58
*Corynebacterium*		3		1	4	
*Cloacibacterium*	2	1			4	20
*Micrococcus*	2	3			2	59
*Comamonas*	3	4				19, 20
*Bradyrhizobium*	4	3				
*Rheinheimera*	2	2			2	
*Lactococcus*	2		2	2		60, 61
*Methylotenera*	3	3				62
*Sphingomonas*	4	2				
*Propionibacterium*		2			4	
*Limnohabitans*	1	2			2	
Staphylococcus	1			1	3	63
*Enterococcus*	1			4		64
*Aerococcus*	2	2			1	
*Parvibaculum*	4	1				
*Acidovorax*	3	1				
*Hydrogenophaga*	4					
*Hyphomicrobium*	4					
Mycobacterium	4					65
*Phenylobacterium*	4					66
*Sediminibacterium*	4					
*Gallibacterium*		4				67, 68
*Hylemonella*		4				69
*Lactobacillus*				4		60, 61
Streptococcus	1	1			1	70
Campylobacter	1			1	1	71, 72
*Clostridium*	3					73
*Enhydrobacter*	3					74
*Methylibium*	3					
*Planctomyces*	3					
*Cupriavidus*		2			1	
*Psychrobacter*	1			1		75
*Aminobacter*	2					
*Janthinobacterium*	2					76
*Legionella*	2					76, 77
*Bacillus*		1		1		
*Curtobacterium*		1		1		78
*Leucobacter*		2				79
*Rothia*		2				80
*Agrobacterium*				2		
*Rickettsiella*				2		
*Exiguobacterium*	1					
*Novosphingobium*	1					
*Sphingobacterium*	1					61
*Anaerococcus*		1				
*Brevundimonas*		1				78
*Kingella*		1				81
*Nevskia*		1				
*Balneimonas*				1		
*Kocuria*					1	82
*Sphingopyxis*					1	

a Values indicate how many (of four) pairwise comparisons where the genus contained differentially abundant ASVs overrepresented in that body site. “Relevant studies” are papers where specific genera have been found to be significant in one of the tested body sites.

### Pagel’s lambda.

To assess whether traits contained phylogenetic signal, Pagel’s lambda was calculated for bird size as a general positive control and for each alpha-diversity metric within each body site. As expected, bird size was always significantly different from 0 (*P < *0.0001) and lambda was between 0.9 and 1.0 (Table S5). For microbial diversity, lambda was significantly different from zero (adjusted *P* [*P*_adj_] < 0.05) four times in the full data set, out of 20 tests: blood-Faith’s PD, buccal-Shannon, intestine-Shannon, intestine-Simpson, and lambda ranged from 0.26 to 0.53 (Table 2). Lambda was significantly different from zero one time in the *Passeriformes*-only data set (out of 16 tests): buccal-observed; lambda was 0.91. Lambda was significantly different from zero two times in data sets containing “*n* > 2” individuals from each species (out of 16 tests): blood-observed, blood-Faith’s PD, and lambda ranged from 0.63 to 0.64.

**TABLE 2:** Regression, phylogenetic generalized least squares, and Pagel’s lambda for the various body sites using all data and two subsets of the data^*a*^

	Non-PGLS			Non-PGLS (log)			PGLS			PGLS (log)			Lambda		
	*R*	*P*	*P*_adj_	*R*	*P*	*P*_adj_	Value	*P*	*P*_adj_	Value	*P*	*P*_adj_	λ	*P*	*P*_adj_
Full dataset									** **			** **			
Blood (*n* = 606 individuals, 187 species)									** **			** **			
Observed	−0.003	0.97	1	0.041	0.58	1	−0.045	0	**0.001**	−20.729	0.006	**0.026**	0.294	0.032	0.126
Faith’s PD	−0.018	0.8	1	0.059	0.43	1	0.005	0	**0**	−2.427	0.003	**0.013**	0.4	0.003	**0.013**
Shannon	−0.006	0.94	1	0.093	0.21	0.84	−0.001	0	**0**	−0.350	0.001	**0.006**	0.254	0.019	0.075
Simpson	−0.046	0.53	1	0.04	0.59	1	0	0	**0**	−0.103	0	**0**	0.041	0.606	1
Buccal (*n* = 346 individuals, 132 species)															
Observed	−0.048	0.59	1	−0.025	0.78	1	−0.023	0.622	1	−17.531	0.507	1	0.228	0.227	0.907
Faith’s PD	−0.810	0.36	1	−0.018	0.83	1	−0.002	0.383	1	−0.900	0.545	1	0.27	0.095	0.38
Shannon	−0.082	0.35	1	−0.130	0.14	0.56	0	0.177	0.708	−0.427	0.01	**0.042**	0.53	0	**0.001**
Simpson	0.019	0.83	1	−0.060	0.5	1	0	0.764	1	−0.046	0.13	0.521	0.363	0.123	0.491
Gizzard (43 individuals, 32 species)															
Observed	−0.240	0.19	0.76	−0.250	0.17	0.68	−0.042	0.437	1	−93.680	0.184	0.736	0	1	1
Faith’s PD	−0.250	0.16	0.64	−0.260	0.15	0.6	−0.004	0.332	1	−9.285	0.047	0.187	0	1	1
Shannon	−0.330	0.061	0.244	−0.240	0.19	0.76	0	0.237	1	−1.223	0.011	**0.045**	0.241	0.583	1
Simpson	−0.300	0.093	0.372	−0.140	0.16	0.64	0	0.681	1	−0.266	0.045	0.181	0	1	1
Intestine (*n* = 200 individuals, 115 species)												** **			
Observed	−0.100	0.28	1	0.032	0.73	1	−0.030	0.692	1	68.327	0.001	**0.005**	0.316	0.161	0.643
Faith’s PD	−0.120	0.22	0.88	0.041	0.67	1	−0.001	0.816	1	6.245	0	**0.001**	0.156	0.361	1
Shannon	−0.066	0.48	1	0.079	0.4	1	0.003	0.705	1	0.785	0	**0.001**	0.351	0.001	**0.005**
Simpson	−0.014	0.88	1	0.092	0.33	1	0.002	0.286	1	0.236	0	**0**	0.267	0.004	**0.015**
Cloaca (*n* = 353 samples, 133 species)															
Observed	0.016	0.86	1	0.05	0.57	1	0.001	0.932	1	8.413	0.45	1	0.339	0.077	0.309
Faith’s PD	0.047	0.59	1	0.074	0.4	1	0.001	0.608	1	1.011	0.281	1	0.383	0.047	0.189
Shannon	−0.150	0.082	0.328	−0.120	0.16	0.64	0	0.225	0.9	−0.134	0.418	1	0.279	0.197	0.788
Simpson	−0.170	0.048	0.192	−0.120	0.18	0.72	0	0.263	1	−0.009	0.805	1	0.165	1	1
*Passeriformes* Dataset												** **			
Blood (*n* = 505 individuals, 130 species)												** **			
Observed	−0.039	0.66	1	0.013	0.89	1	−0.006	0.861	1	−32.907	0.006	**0.022**	0	1	1
Faith’s PD	−0.037	0.68	1	0.05	0.57	1	−0.001	0.837	1	−4.489	0.001	**0.005**	0	1	1
Shannon	0.03	0.74	1	0.051	0.57	1	0	0.749	1	−0.872	0	**0**	0	1	1
Simpson	0.053	0.55	1	0.069	0.43	1	0	0.688	1	−0.197	0.001	**0.002**	0	1	1
Buccal (*n* = 272 individuals, 86 species)															** **
Observed	0.13	0.23	0.92	0.13	0.23	0.92	0.611	0.362	1	9.115	0.805	1	0.919	0.001	**0.003**
Faith’s PD	0.18	0.095	0.38	0.17	0.12	0.48	0.039	0.415	1	−0.114	0.965	1	0.564	0.132	0.528
Shannon	0.19	0.083	0.332	0.17	0.11	0.44	0.005	0.442	1	−0.072	0.842	1	0.333	0.021	0.083
Simpson	0.091	0.41	1	0.072	0.51	1	0	0.834	1	−0.047	0.463	1	0	1	1
Intestine (*n* = 151 individuals, 79 species)															
Observed	−0.032	0.78	1	0.06	0.6	1	−0.014	0.956	1	6.432	0.874	1	0	1	1
Faith’s PD	−0.021	0.85	1	0.088	0.44	1	0.002	0.926	1	1.044	0.751	1	0	1	1
Shannon	−0.026	0.82	1	0.012	0.92	1	−0.001	0.718	1	−0.251	0.552	1	0	1	1
Simpson	−0.001	0.99	1	−0.016	0.89	1	0	0.889	1	−0.046	0.649	1	0.05	1	1
Cloaca (*n* = 275 samples, 85 species)															
Observed	−0.048	0.66	1	−0.017	0.87	1	−0.954	0.044	0.177	−46.868	0.057	0.227	0.582	0.022	0.086
Faith’s PD	−0.042	0.7	1	−0.009	0.93	1	−0.082	0.038	0.154	−4.345	0.033	0.134	0.554	0.032	0.13
Shannon	−0.250	0.022	0.088	−0.180	0.092	0.368	−0.042	0	**0**	−2.252	0	**0**	0.206	0.185	0.741
Simpson	−0.200	0.062	0.248	−0.150	0.16	0.64	−0.008	0	**0**	−0.428	0	**0**	0.115	0.534	1
Only species with >2 individuals									** **			** **			** **
Blood (*n* = 453 individuals, 64 species)									** **			** **			** **
Observed	0.041	0.75	1	0.2	0.12	0.48	−0.071	0	**0**	−8.308	0.001	**0.002**	0.643	0.005	**0.02**
Faith’s PD	0.085	0.51	1	0.26	**0.04**	0.16	−0.007	0	**0**	−0.811	0	**0**	0.636	0.002	**0.007**
Shannon	0.15	0.23	0.92	0.23	0.067	0.268	−0.001	0	**0**	−0.117	0	**0**	0.389	0.217	0.868
Simpson	0.17	0.19	0.76	0.18	0.16	0.64	0	0	**0**	−0.034	0	**0**	0	1	1
Buccal (*n* = 222 individuals, 41 species)															
Observed	0.2	0.23	0.92	0.11	0.52	1	0.113	0.024	0.098	−1.575	0.11	0.44	0	1	1
Faith’s PD	0.23	0.17	0.68	0.12	0.48	1	0.007	0.001	**0.006**	−1.486	0.011	**0.045**	0	1	1
Shannon	0.05	0.77	1	0.14	0.41	1	−0.001	0	**0**	−0.262	0.006	**0.023**	0.521	0.059	0.236
Simpson	0.064	0.71	1	0.15	0.4	1	0	0	**0**	−0.054	0	**0**	0.515	0.209	0.837
Intestine (*n* = 71 individuals, 18 species)															
Observed	0.31	0.21	0.84	0.16	0.53	1	1.736	0.294	1	85.404	0.761	1	0.751	0.342	1
Faith’s PD	0.28	0.27	1	0.15	0.56	1	0.136	0.188	0.75	7.305	0.646	1	0	1	1
Shannon	0.18	0.46	1	0.04	0.88	1	0.005	0.071	0.285	0.355	0.299	1	0.943	0.112	0.447
Simpson	0.11	0.66	1	−0.014	0.96	1	0	0.024	0.094	0.051	0.15	0.599	0.759	0.331	1
Cloaca (*n* = 231 samples, 44 species)															
Observed	0.19	0.24	0.96	0.021	0.9	1	0.047	0.303	1	7.31	0.436	1	0	1	1
Faith’s PD	0.25	0.13	0.52	0.093	0.57	1	0.004	0.093	0.37	1.096	0.253	1	0	1	1
Shannon	0.18	0.27	1	−0.011	0.95	1	0	0.011	**0.043**	0.119	0.076	0.304	0	1	1
Simpson	0.14	0.38	1	0	1	1	0	0.003	**0.012**	0.005	0.007	**0.03**	0	1	1

a *Passeriformes*-only and only species with more than two individuals represented *n* > 2. Statistics were run for both the raw and log weight of the host. Bold represents an adjusted *P* value <0.05.

### Phylogenetic generalized least squares.

**FIG 5:**
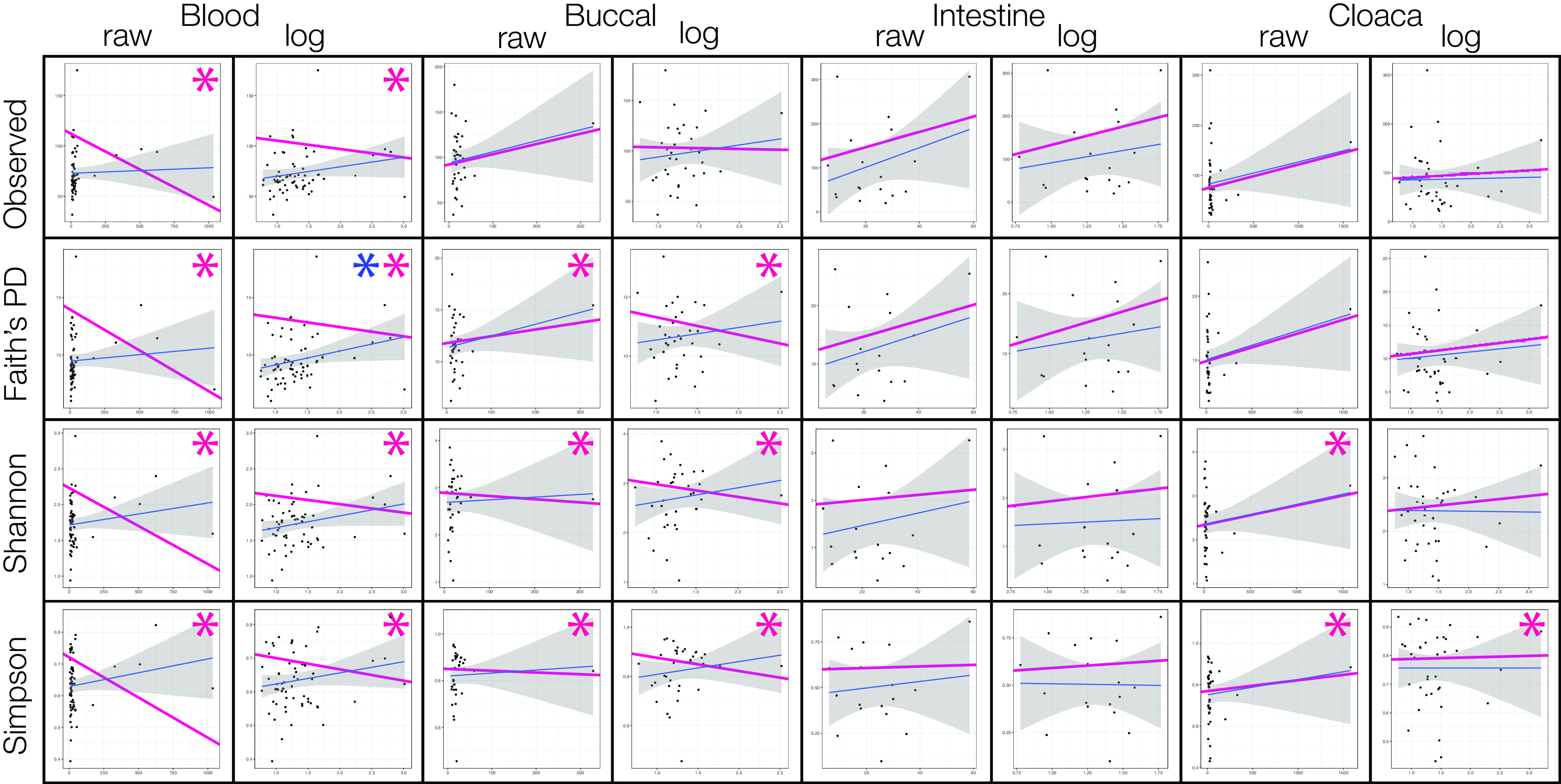
Linear regressions (blue line with gray confidence intervals) and phylogenetic generalized least-squares (pink line) for the species with *n* > 2 data set: four body sites (blood, buccal, intestine, cloaca) and both raw weight and log transformed weight results shown for all four alpha-diversity and metrics (observed ASVs, Faith’s PD, Shannon, and Simpson). Blue and pink asterisks indicate an adjusted *P* value <0.05 for the regular and PGLS regressions, respectively.

Diversity metrics for samples at each body site that belonged to the same species were averaged to produce one diversity measurement per species for each of the four alpha-diversity metrics. In the full data set, none of the linear (nonphylogenetically controlled) regressions were significant (Table 2). Using phylogenetic generalized least squares (PGLS) on the full data set, all four alpha-diversity metrics for the blood were significantly associated with body size, in raw and log-transformed comparisons (*P*_adj_ < 0.05); all other comparisons were not significant. These relationships showed a negative correlation. In the log-transformed tests, the buccal-Shannon (negative correlation), the gizzard-Shannon (negative correlation), and all four comparisons within the intestines (positive correlation) were significant. When looking within the *Passeriformes*-only data set, all four blood comparisons in the log-transformed tests were significant (negative correlation) and the Shannon and Simpson tests at the cloaca were significant as well (negative correlations). All other tests within the *Passeriformes*-only data set were not significant. Using only species with *n* > 2 individuals, blood was significant for all comparisons, in both raw and log-transformed calculations; buccal comparisons using Faith’s PD (positive correlation for raw, negative correlation for log-transformed), Shannon (negative correlation), and Simpson (negative correlation) were also significant in both raw and log-transformed data (Fig. 5). Cloaca tests using raw weights were significant using Shannon and Simpson diversity metrics and Simpson metric only with the log-transformed data (all positive correlations).

### Phylosymbiosis.

**FIG 6:**
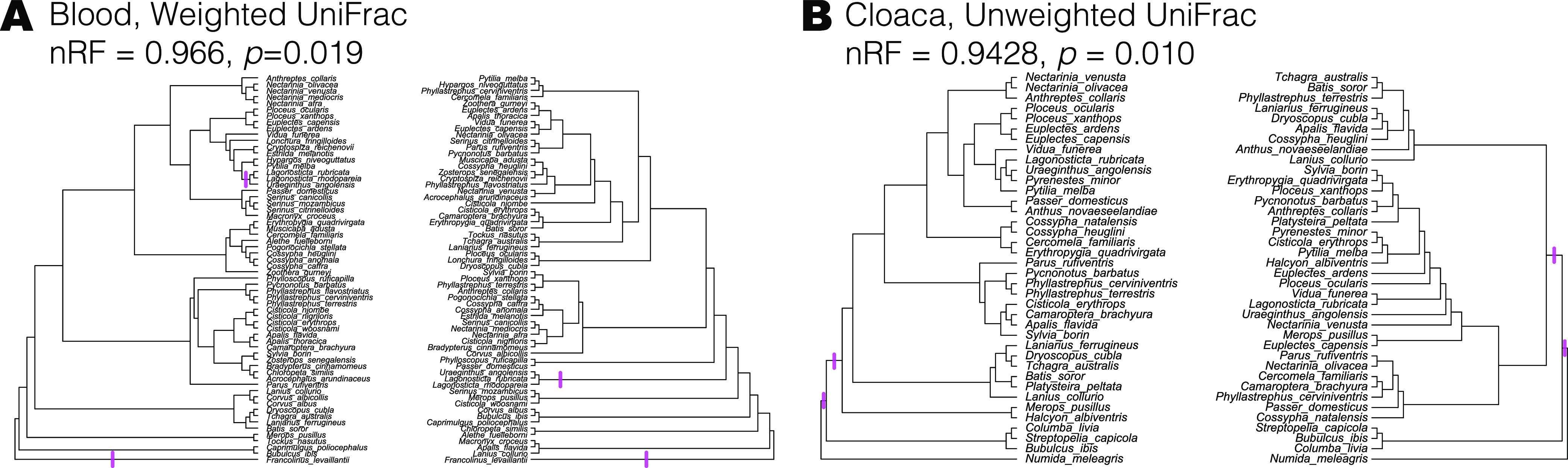
Phylosymbiosis of bird microbiota was weak for all comparisons (shown in Fig. S6). Two of the eight comparisons were significantly similar: in the blood samples, there were two congruent “splits” in the bird phylogeny (left) and dendrogram of weighted UniFrac distances (right) (A) and in the cloaca samples, which also had two congruent splits in the bird phylogeny (left) and unweighted UniFrac dendrogram (right) (B).

Eight tests for phylosymbiosis were conducted, using both weighted and unweighted UniFrac on four body sites (blood, buccal, intestine, and cloaca), including only species with *n* > 2 individuals (Table 3). Two of these tests were significant using the permutation test (Fig. 6; Fig. S6): blood using weighted UniFrac and cloaca using unweighted UniFrac. Notably, both these microbiota dendrograms contained only two partitions that were congruent with the host phylogeny. In other comparisons, either one or zero partitions were congruent (Fig. S6).

**TABLE 3:** Calculations of phylosymbiosis for the four body sites in the species with >2 sample data set, using both weighted and unweighted UniFrac distances^*a*^

Body site/UniFrac distance	*n*	nRF	*P*
Blood			
Weighted	62	0.9661	**0.02**
Unweighted	62	0.9831	0.16
Buccal			
Weighted	34	0.9677	0.17
Unweighted	34	1.0000	1
Intestine			
Weighted	18	0.9333	0.22
Unweighted	18	1.0000	1
Cloaca			
Weighted	38	0.9714	0.16
Unweighted	38	0.9429	**0.01**

a *P*-values less than 0.05 are bolded.

## DISCUSSION

Biological understanding of vertebrates is incomplete without knowledge of the microbiome. One of the specific goals of evolutionary biology is to discover and describe biodiversity (see reference 17); herein, 1,736 samples from 779 wild birds have expanded our knowledge of birds and their associated microbes. This data set, assessing microbiota from over 200 host species, provides a fundamental description of the microbiomes of diverse and previously undescribed body sites. In addition to characterizing the diversity of the microbiota associated with specific body sites, we assess the phylogenetic signal within the microbiota of these birds in several ways. Our results indicate a variable influence of host phylogeny on the composition of the microbiota, depending on the body site characterized. Additionally, we see the variable influence of specific host ecological or behavioral traits on the diversity and abundance of bacterial communities.

Significant variation in microbial community composition has been previously observed across anatomical sites on a host’s body, including within distinct sections of the gastrointestinal tract (12, 15) and a hypothesized role for particular gastrointestinal organs (18). This is unsurprising, as distinct body sites within a host often encompass unique environments in which certain microbes thrive while others are unable to establish a population. Our results align with this by identifying distinct variation across five unique body sites. Additionally, within specific body sites, our alpha- and beta-diversity results generally align with previous research. The cloacal and intestinal microbiomes are consistent with previous studies that show a dominance of *Proteobacteria* and *Firmicutes* (reviewed in reference 14). The blood and buccal results are similar at the phylum level to those of previous avian studies (19, 20), though some differences exist. For example, the blood samples in this study have nearly 20% more *Proteobacteria* on average than Mandal et al. (20). However, such differences may be unsurprising given that wild birds tend to host higher percentages of *Proteobacteria* (14) and our results derive from many species.

Alpha diversities exhibited various patterns, depending on body site and the diversity metric applied. Few consistent patterns emerged. One exception to this is that the buccal microbiota exhibited significantly higher alpha diversity than the cloacal microbiota, when comparing within the full data set and with the majority of intraspecific tests. This is notable as both sites are “ends” of the gastrointestinal tract and come into contact with similar external environments. One hypothesis for the higher diversity in buccal cavities is that they are more likely to be exposed to environmental bacteria through the consumption of food materials, preening, or general contact with the environment, such as through holding nesting materials in the beak. As these environmental microbes are ingested, they may slowly become reduced in abundance as they pass through the gastrointestinal tract, as suggested in the comparison between buccal and cloacal microbiota of great tits (19).

Generally, body sites along the gastrointestinal tract, such as the gizzard or intestines, exhibited higher diversity compared to the blood microbiota. Our expectation was that blood would exhibit lower diversity, which was generally but not always the case. Blood has long been thought of as a sterile tissue. The existence of a true blood microbiota has been contentious (e.g., reference 21). Despite this, we recovered a distinct microbiota from blood samples, and there are several possible explanations for this. First, 16S amplicon sequencing does not distinguish between living versus dead bacteria, dormant versus active bacteria, or even intact chromosomes versus pieces of DNA. It is possible that the diversity observed within the blood includes “debris” and is not actually a resident microbiota. Second, because this is a comparative study containing hundreds of host species, species-level properties may be complicating the interpretation of the results. Third, the biggest drawback of this study is the lack of negative controls from which we could estimate contaminants, so we cannot directly rule out widespread contamination, which may be especially influential in blood samples. Low biomass samples are prone to larger effects of contamination (22), and blood samples may fit this designation; we therefore acknowledge that some of the counterintuitive results observed in our analyses may be the result of or influenced by contamination. However, we believe there is supporting evidence for this not being the case. Many of the taxa found in each of our body sites were previously identified in avian microbiomes (see reference citations in Table 1). It is also unlikely that contamination would result in a phylogenetic correlation between body size and microbiota of the blood samples (and no other body site). Counter to the longstanding idea that blood is a sterile environment, recent studies have demonstrated that circulating blood does contain a unique microbiota (22) that, similar to gastrointestinal microbiota, can be influenced by specific host characteristics such as diet (23), disease status (24), or geographic location (25). We suggest that blood microbiota is influenced by host phylogeny but it is certainly true that the avian blood microbiome requires additional tests to characterize and understand it.

The majority of our study focused on samples collected from five body sites: blood, buccal, gizzard, lower intestines, and cloaca. These sample types were significantly different from each other (*P < *0.001), and the variation explained by body site was relatively high (6.7% to 17.0%). These measurements are lower than some other bird body site studies (15), but this is perhaps to be expected as each sample set in this study incorporates dozens to hundreds of bird species that may have distinct microbiomes. When the body sites were analyzed separately, the correlation of bird taxonomy (order, family, and genus) to microbiota diversity was high and significant for all taxonomic ranks tested (*P < *0.001); effect sizes increased from order to genus, but this is to be expected, as the data are being divided into more groups as taxonomic resolution increases (i.e., degrees of freedom increase). The ecological variables also tended to be significant, although the effect sizes of diet, nesting, and social behaviors were lower than that of host taxonomy and always less than 10%. In many cases, if a variable was found to be significantly correlated with the microbiota, the difference in group dispersions was also significant (Table S4), violating the assumption of homoscedasticity (i.e., if two groups have significantly different dispersions, the significance of the original test is questionable because it is influenced by the dispersions). The correlation between plate name and the microbiota was also significant and the effect sizes varied from 3 to 17%. These batch effects may have been exacerbated due to the nonrandom placement of samples on the sequencing plates, since sequencing plates may contain multiple samples from closely related birds. Notably, host variation in community composition between male and female hosts was never significant for any of the body sites or metrics. Although some variation between males and females has been observed during breeding periods, more often the differences are minor/insignificant (e.g., reference 26).

Many ornithologists are interested in studying the microbiome without harming the bird, and therefore many have asked whether nondestructive sampling (e.g., buccal swabs, cloacal swabs, feces) is adequate to describe the gut microbiome (27). We compared cloacal and intestine samples and found substantial and roughly equivalent unique components for each sample type at the ASV level; however, at the level of the sequencing reads, almost all the diversity was shared (Fig. S5). Therefore, the unique ASVs contribute far less to the total microbiota than the shared ASVs. Compositionally, 56 genera contained ASVs that were differentially expressed across body sites. These genera may be important to the respective body sites where they are differentially expressed and deserve targeted inquiry into their functional roles in the microbiome. Importantly, the collection of the body site samples was inconsistent: blood was stored on Whatman paper, and buccal and cloaca samples were collected using sterile swabs, and gizzard and intestine consisted of tissue biopsy specimens. Therefore, significant pairwise differences across these sampling types may reflect bias in the sampling method. Finally, because each body site contained many species and samples may have been collected at different times of the year, there may be species differences or seasonal effects confounding the results. We believe the low sample sizes and use of many species minimizes, but does not eliminate, this possibility.

The microbiome is a trait of the host that may not be independent of the underlying phylogeny. Strictly speaking, phylogeny describes the evolutionary history of organisms but for a variety of reasons, phylogeny can capture more than just evolutionary history and more closely related organisms frequently have more similar traits than more distantly related organisms (“phylogenetic signal”). Therefore, comparisons of microbial “traits” across species need to control for the phylogeny of the hosts.

Pagel’s lambda showed an inconsistent (across body sites) and low level of phylogenetic signal in the microbiota diversity metrics. These results may not be entirely surprising as previous comparative work using 16S rRNA amplicons found that a white noise model (of no phylogenetic signal) may fit the avian microbiome better than a neutral model or a model that includes selection (28). This indicates that microbiota may contain little to no phylogentic signal.

In exploring the relationship between host size and the richness and diversity of the microbiota, we found significant correlations when using phylogenetic comparative methods (Table 2), although many of these associations were not in the expected direction. In the full data set, blood samples show a significant negative correlation between their microbial diversity and the average host size for all four diversity metrics, indicating that, when phylogeny is taken into account, larger birds have a lower than expected diversity in their blood. This finding supports the hypothesis that a higher abundance of circulatory immune cells may restrict microbial diversity in the blood, leading to a negative correlation between host size and blood microbiota diversity. Further specific tests are required to thoroughly test this hypothesis.

Because associations with life history traits vary within and across bird orders (29), we restricted the additional analysis to the most represented host order (*Passeriformes*) and reran the PGLS analyses. All four of the microbiome metrics for the cloacal samples exhibited significant negative correlations with the raw weight of the birds and three were significant with the log-transformed weights (*P*_adj_ < 0.05), These results support the findings of Bodawatta et al. (30) that investigated the cloacal microbiome of Passerines and found highly variable species-level microbiota. diversity-area relationships predict that larger areas will house more diversity, but many of our results appear to show the opposite: either a significant negative correlation or no correlation at all. Because the role of a nontypical individual could heavily influence results in a comparative context, we also subset our body site data sets to only contain species with *n* > 2 individuals. Blood was again negatively correlated with bird size in all tests. Although avian blood microbiota does not appear to adhere to the Diversity-Area and species-area relationships, there is an alternative explanation. Larger birds may produce a higher immune cell output than smaller birds (16), such that larger birds may be more capable of inhibiting the establishment of new microbial colonizers.

Buccal samples were significant in three out of four metrics for both raw and log-transformed weight (with one association being positive and the other five being negative). Three cloaca tests were also significant and positive. Each significant test shows a strong relationship between bird size and microbial diversity that is not explained by phylogeny, but any inconsistency of results across common diversity metrics undermines the strength of the significant results. Therefore, two methodological caveats must be acknowledged: the analyses were only conducted on a single starting tree and our sampling across species was generally uneven.

The lack of consistency in the phylogenetic comparative tests and metrics, the low levels of phylosymbiosis, and the large variation in composition and alpha diversity in the avian microbiome may not be surprising. The avian microbiome appears to contain significant variation on an individual basis (30) and the role of environmental and transient microbes may be large. It is possible that there is too much variation on an individual (or a day to day) basis for meaningful patterns to be drawn from avian microbiota. The exception appears to be the blood microbiota, which were significantly associated with bird size in 20 out of 24 tests and were always negative. Additional tests, inclusion of functional datatypes (metagenomics, metatranscriptomics, proteomics), and hypothesis-based experiments are required to continue to better understand the avian microbiome.

## MATERIALS AND METHODS

### Sample collection and sequence processing.

Sampling was conducted as previously described (31). Briefly, adult birds and their associated tissue specimens were collected for museum preservation from October to November 2009 and February to March 2011 in Malawi, detailed in Table S2. Blood samples were obtained via brachial venipunctures and stored on Whatman FTA cards. Bird skin was sterilized with an ethanol swab prior to brachial venipuncture and a sterile needle was used for each individual. Approximately 30 μL of blood was transferred to Whatman FTA cards using sterile hematocrit tubes. Buccal and cloacal samples were collected by swabbing with sterile cotton swabs and cryopreserved in liquid nitrogen. Liver, spleen, and lower intestinal tracts were dissected from euthanized animals and cryopreserved in liquid nitrogen; the total number of liver and spleen samples were too low to be included in comparative analyses but are included in the supplemental material for descriptive purposes. Although field conditions were not sterile by laboratory standards, field crews were cognizant of potential contamination and took appropriate measures to avoid contamination, including sterilization of all tools between uses. All voucher specimens and their associated tissue specimens are housed at the Field Museum of Natural History in Chicago, IL, USA.

All samples were processed following the Earth Microbiome Project (EMP) standard 16S rRNA metabarcoding protocols (32–34). To reduce the possibility of cross-contamination between different sample types, samples from each body site (e.g., blood, intestines, etc.) were processed with only samples from the same body site from DNA extraction until sequencing. DNA was extracted using PowerSoil PowerMag DNA extraction kits (Qiagen). Amplification was performed in triplicate and used the 515f and 806r EMP primers targeting the V4 region of the 16S rRNA gene. Amplicons from individual samples were pooled and normalized for sequencing of paired-end 150 bp reads. All sample types were sequenced on the Illumina MiSeq platform. Sequence data were uploaded to the QIITA web-based microbiome analysis platform for initial processing (made available previously via QIITA ID 11166 by reference 35).

Raw sequences were demultiplexed and quality filtered using the QIIME2 pipeline (36) and forward and reverse reads were joined and processed using Deblur to remove sequencing errors and refine sequences to the high-quality, unique sequencing reads or ASVs (37). Bacterial taxonomy was assigned using the QIIME2 naive Bayes feature-classifier against Greengenes 13_8 reference database (38); reads that aligned to chloroplasts, archaea, or nonbacterial lineages or that were unassigned at the phylum level were removed.

### Statistical analysis.

**(i) Rarefaction.** Statistical analyses were conducted using R version 4.1.1 (39), primarily using the phyloseq package (40). We produced rarefaction curves to identify sufficient sequencing depth using the vegan package (41). For initial analyses, all samples were combined and rarefied to 4,000 sequences per sample, leading to 1,606 samples. For body-site specific analyses, samples were subset by body site, and then rarefied (blood: 4,030 sequences; buccal: 10,961 sequences; cloaca: 10,329 sequences; intestines: 9,094 sequences; and gizzard: 22,306 sequences). Rarefaction curves are available in the supplemental material (Fig. S1).

**(ii) Alpha diversity.** Alpha diversity was calculated using the observed number of ASVs, Shannon diversity index (42), Simpson diversity index (43), and Faith’s PD (44); these were calculated using the phyloseq and picante (45) packages in R. We chose these metrics to represent diversity in multiple ways to draw a holistic picture of microbiota diversity. Pairwise comparisons using a *t* test between body sites within each measure of alpha diversity to identify significant differences. To assess alpha diversity within host species, we compared four diversity metrics of three body sites (blood, buccal, cloaca) from 12 species in which at least seven samples were collected from each of the blood, buccal, and cloacal body sites.

**(iii) Beta diversity.** Beta diversity was calculated across numerous host variables and body sites using weighted and unweighted UniFrac metrics (45) on a phylogeny produced via the alignment of ASVs and construction of a Neighbor-Joining tree using the program FastTree (46). Ordinations were viewed using nonmetric multidimensional scaling (NMDS). Statistical significance of categorical variables was calculated with PERMANOVA using the adonis2 function and differences in the dispersion of groups was calculated using the betadisp function in the vegan package (41, 47). A *P* value of 0.05 was considered significant for all beta-diversity comparisons. The significance of body site was tested in the full data set and then multiple host variables were tested within the subset body sites, including bird taxonomic level (order, family, genus), diet (animal material, plant material, omnivore), nest type (open cup, closed cup, closed cup parasite, open cup parasite, cavity), nest location (understory, variable, ground, canopy or subcanopy, cliff or bank), nesting habits (solitary, parasitic, colonial), social system (solitary or pairs, single species flock, mixed species flock), and sex (male or female). To assess the batch effect within body site samples, we also compared the beta diversity of the extraction plate.

**(iv) Differential abundance.** DESeq2 (48) was used to identify bacterial taxa that may be unique to one or conserved across multiple body sites (buccal, cloacal, intestines, gizzard). For pairwise comparisons, we rarefied all body-site libraries to the lower number used between the two sites (i.e., comparisons between intestinal and Buccal samples were rarefied to 9,094 sequences per sample). For these analyses, an alpha value of 0.01 was used for significance.

(v) **Phylogenetic comparative analyses.** BirdTree.org was used to create phylogenetic trees of host species represented in each body site (49, 50). “Hackett All Species” was used within this tool (51). We used phytools to reconstruct the phylogeny of host species from BirdTree.org (52). To quantify phylogenetic signal in the microbiome, Pagel’s lambda was estimated for the four alpha-diversity metrics (observed ASVs [after rarefying], Faith’s PD, Shannon, and Simpson), using phytools in R and the phylogeny of the species estimated from birdtree.org (52).

To compare two host traits, bird size, and microbiome diversity, while controlling for host phylogeny PGLS was performed independently on each of the rarefied body-site data sets. Weight for each host species was calculated using averaged field measurements of all birds sampled during specimen collection. For species with no recorded weight, the average species weight in Birds of the World (53) was used. Following that, the samples were divided by body site and samples were rarefied as described above. Alpha-diversity metrics were calculated for each species by averaging the alpha diversity of individuals within each species, resulting in one observed richness, one Shannon, one Simpson, and one Faith’s PD value for each species within each body site group.

First, each alpha-diversity metric was plotted against the average species weight without controlling for host phylogeny. Pearson’s correlation coefficient, measuring the linear correlation between two variables, was used to determine the correlation between the two variables with a 95% confidence interval (54). Next the same variables were tested against each other while controlling for phylogeny using a PGLS analysis in R using the package Geiger (55, 56).

Because the phylogenetic signal of bird size is known to vary when analyzing across orders versus within orders (29), each sample type was further subset to include only the largest bird order, *Passeriformes*, referred to as the “*Passeriformes* only” data set. Finally, to minimize the effect of outlier samples, the data set was reduced to only include species with three or more individuals, referred to as the *n* > 2 data set. The gizzard data sets were removed from further analysis due to low sample size (*Passeriformes* only: 9 individuals from 8 species; *n* > 2: 10 individuals from 3 species). Blood, buccal, cloaca, and intestine body site subsets were tested using the same methods as above. For these analyses, *P* values were Bonferroni corrected and an alpha value of 0.05 was used.

**(vi) Phylosymbiosis.** To assess shared patterns of divergence between host phylogeny and microbiome similarity, the phylogeny obtained above was compared to the body site specific data sets that contained only species with more than two individuals represented (*n* > 2). The hclust function was used to create dendrograms from the weighted and unweighted UniFrac distance matrices and the normalized Robinson-Foulds (nRF; reference 57) distance was calculated for the host phylogeny and the microbiome dendrogram. nRF distance can vary from 0, indicating perfect congruence between the two tree topologies, and 1, indicating no congruence between the two tree topologies. A permutation test was used to calculate significance by randomizing the tips on the microbiome dendrogram and then recalculating the nRF distance. This was done 1,000 times and the *P* value was the number of trials out of 1,000 that were equivalent to or more congruent than the empirical nRF distance. For these analyses, an alpha value of 0.05 was used to assess significance.

### Benefits sharing statement.

Benefits generated from this research accrue from the sharing of our data and results on public databases as described above. Physical specimens have been accessioned in biological repositories at the Field Museum of Natural History and the Museums of Malawi through which they are accessible for scientific research.

### Data availability.

For the cloacal and intestinal data: 16S rRNA sequences are publicly available via the QIITA platform under study identifier (ID) 11166 (https://qiita.ucsd.edu/study/description/11166) and the European Bioinformatics Institute (EBI) under accession numbers PRJEB35449. For all other body site data, the QIITA ID is 14978. Metadata files associated with sequence data are available in this study in Table S2. All sequences and metadata are also available per request.
