# Supplementary material for: Body Size Poorly Predicts Host-Associated Microbial Diversity in Wild Birds
Source: Microbiol Spectr. 2023 Apr 11;11(3):e03749-22. doi: 10.1128/spectrum.03749-22 (PMC10269867; doi:10.1128/spectrum.03749-22)

Supplemental Figures for Herder, Skeen, Lutz & Hird, 2023, *Microbiology Spectrum*,
"Body size poorly predicts host-associated microbial diversity in wild birds"

Figure S1. Rarefaction curves for each sample type with red dotted line indicating the number of sequences to which the samples were rarefied: A. blood, B. buccal, C. cloaca, D. intestine, E. gizzard, F. liver, G. spleen.

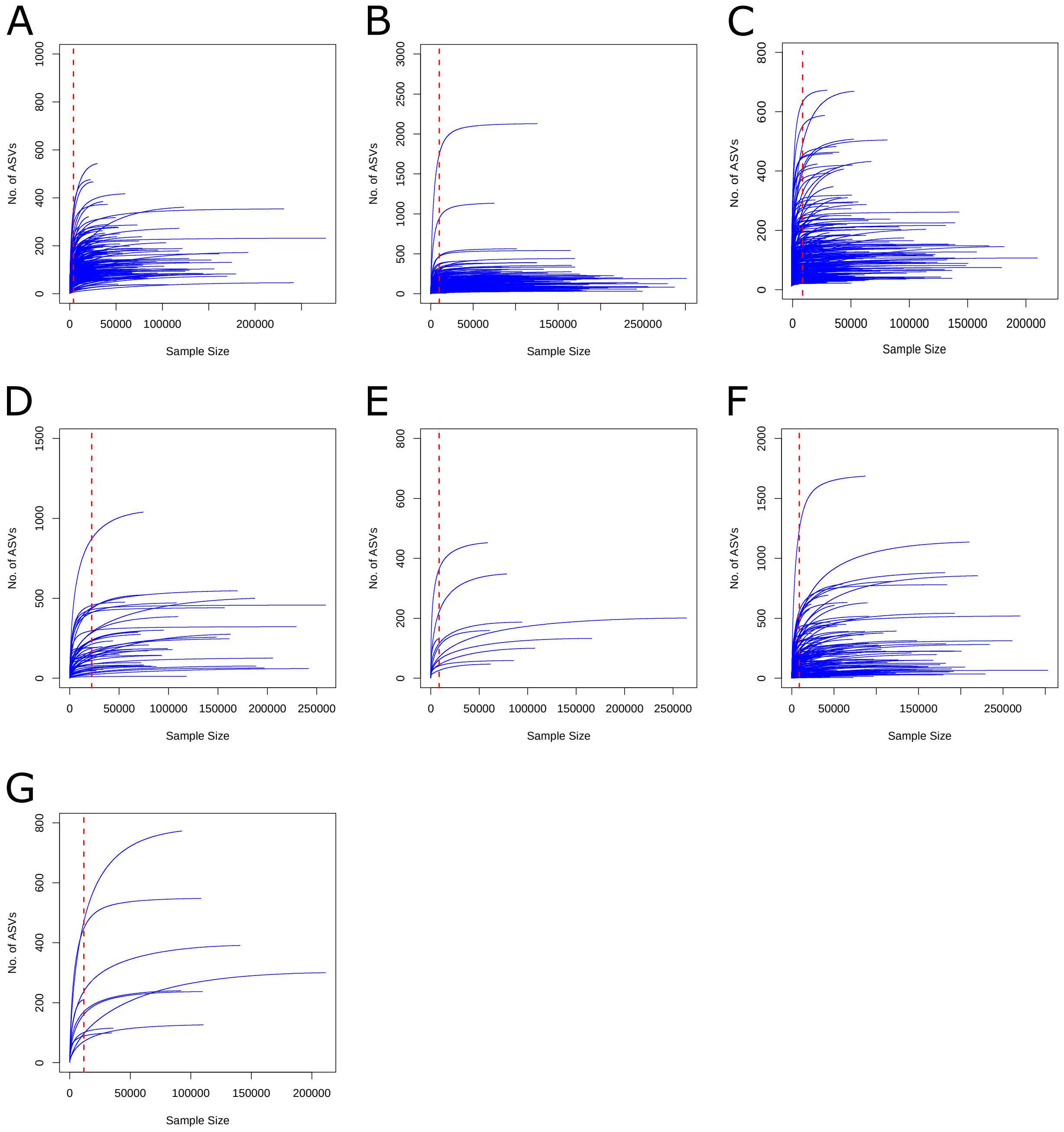

Figure S2. Phylum level relative abundance stacked bar charts of body sites, separated by host order and rarefied to 4,000 reads. The order Passeriformes is further subdivided into family. Bacterial phyla with total abundance less than 1% are summed together and represented by the grey bar. A. blood, B. buccal, C. cloaca, D. gizzard, E. intestine, F. liver, G. spleen.

S2A Blood

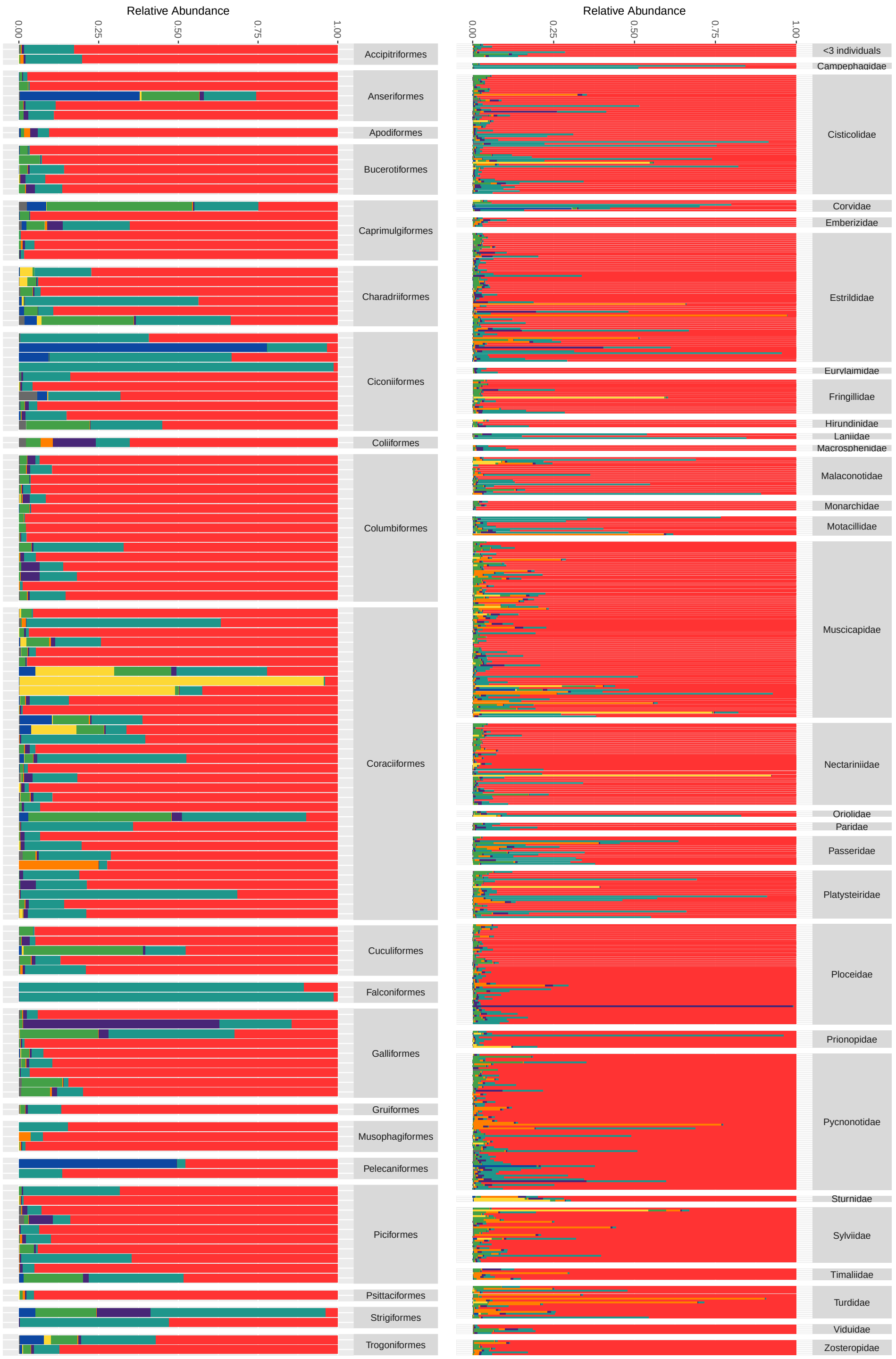

S2B Buccal

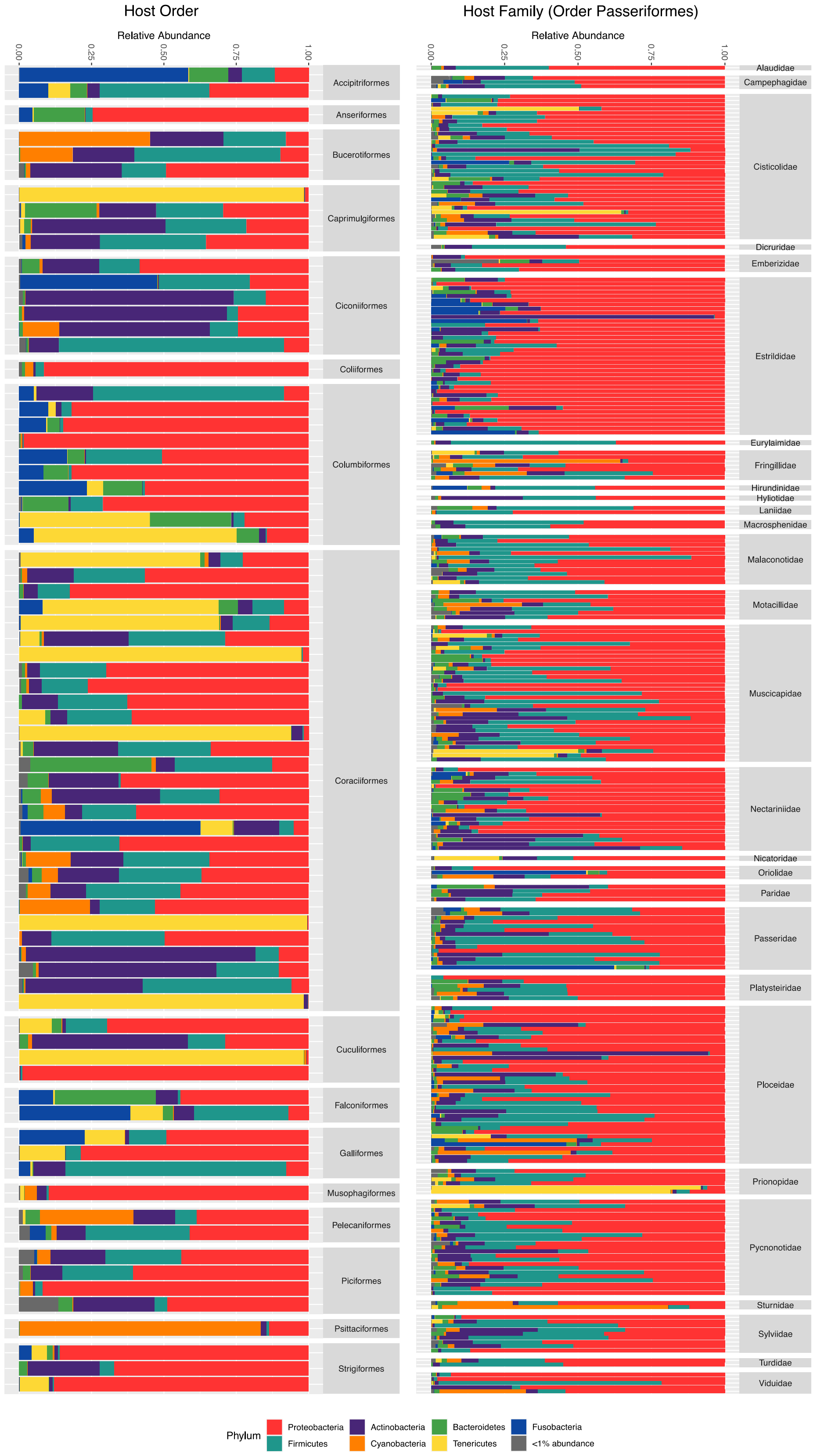
Host Order
Relative Abundance
0.00
0.25
0.50
0.75
1.00
Accipitriformes
Anseriformes
Bucerotiformes
Caprimulgiformes
Ciconiiformes
Coliiformes
Columbiformes
Coraciiformes
Cuculiformes
Falconiformes
Galliformes
Musophagiformes
Pelecaniformes
Piciformes
Psittaciformes
Strigiformes
Host Family (Order Passeriformes)
Relative Abundance
0.00
0.25
0.50
0.75
1.00
Alaudidae
Campephagidae
Cisticolidae
Dicruridae
Emberizidae
Estrildidae
Eurylaimidae
Fringillidae
Hirundinidae
Hylotidae
Laniidae
Macrosphenidae
Malaconotidae
Motacillidae
Muscicapidae
Nectariniidae
Nicatoridae
Oriolidae
Paridae
Passeridae
Platysteiridae
Ploceidae
Prionopidae
Pycnonotidae
Sturnidae
Sylviidae
Turdidae
Viduidae
Phylum
Proteobacteria
Firmicutes
Actinobacteria
Cyanobacteria
Bacteroidetes
Tenericutes
Fusobacteria
<1% abundance

S2C Cloaca

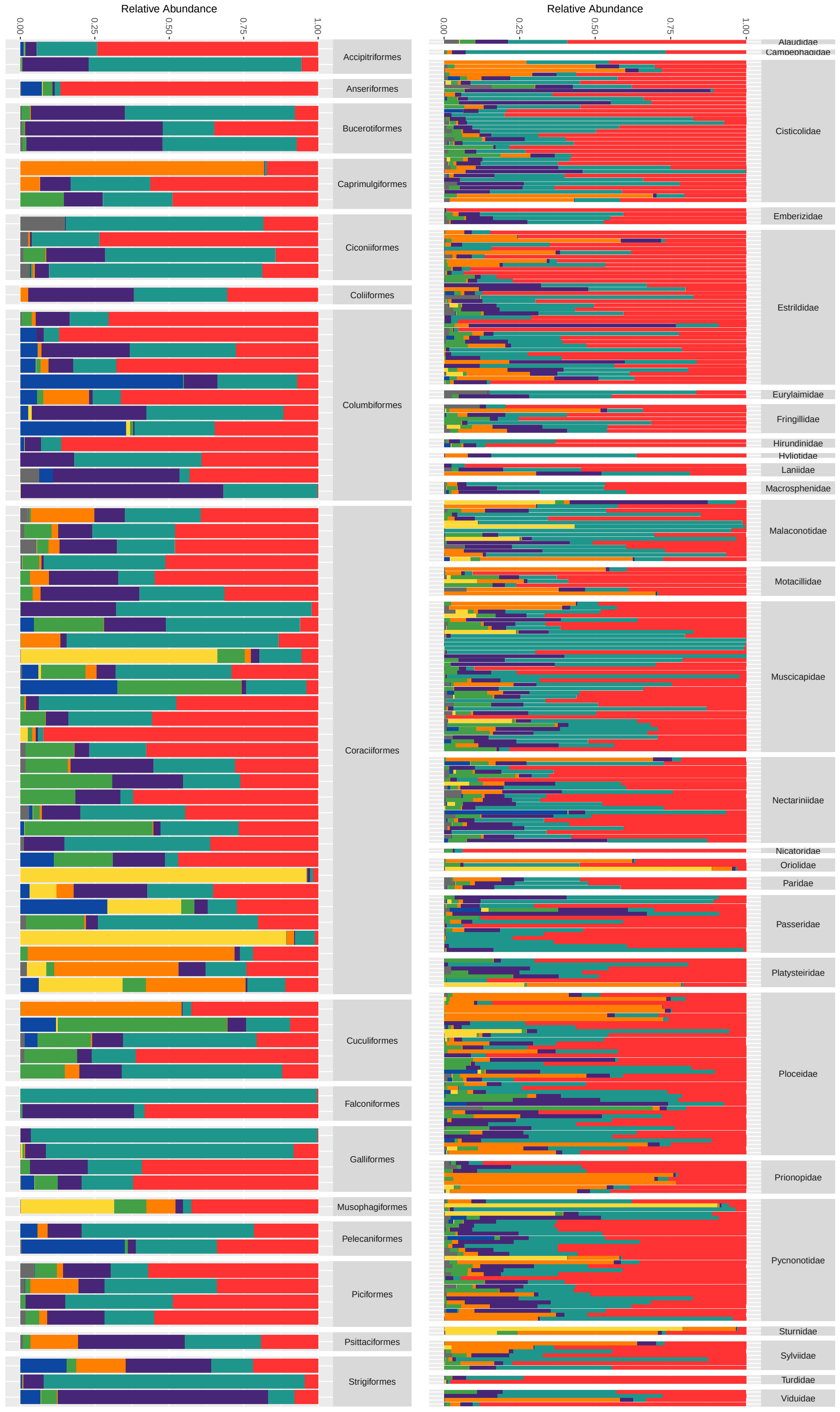
Relative Abundance
0.00
0.25
0.50
0.75
1.00
Accipitriformes
Anseriformes
Bucerotiformes
Caprimulgiformes
Ciconiiformes
Coliiformes
Columbiformes
Coraciiformes
Cuculiformes
Falconiformes
Galliformes
Musophagiformes
Pelecaniformes
Piciformes
Psittaciformes
Strigiformes
Relative Abundance
0.00
0.25
0.50
0.75
1.00
Alaudidae
Campephagidae
Cisticolidae
Emberizidae
Estrildidae
Eurylaimidae
Fringillidae
Hirundinidae
Hyliotidae
Laniidae
Macrosphenidae
Malaconotidae
Motacillidae
Muscicapidae
Nectariniidae
Nicatoridae
Oriolidae
Paridae
Passeridae
Platysteiridae
Ploceidae
Prionopidae
Pycnonotidae
Sturnidae
Sylviidae
Turdidae
Viduidae

S2D Gizzard

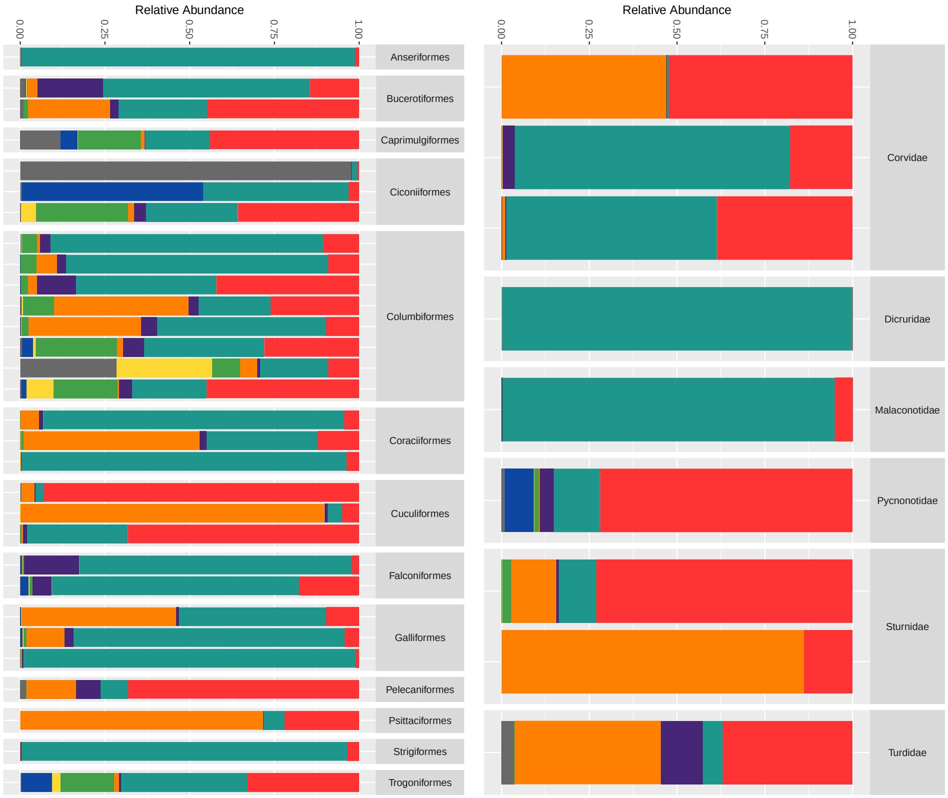

Relative Abundance
0.00
0.25
0.50
0.75
1.00
Anseriformes
Bucerotiformes
Caprimulgiformes
Ciconiiformes
Columbiformes
Coraciiformes
Cuculiformes
Falconiformes
Galliformes
Pelecaniformes
Psittaciformes
Strigiformes
Trogoniformes
Relative Abundance
0.00
0.25
0.50
0.75
1.00
Corvidae
Dicruridae
Malaconotidae
Pycnonotidae
Sturnidae
Turdidae

S2E Intestines

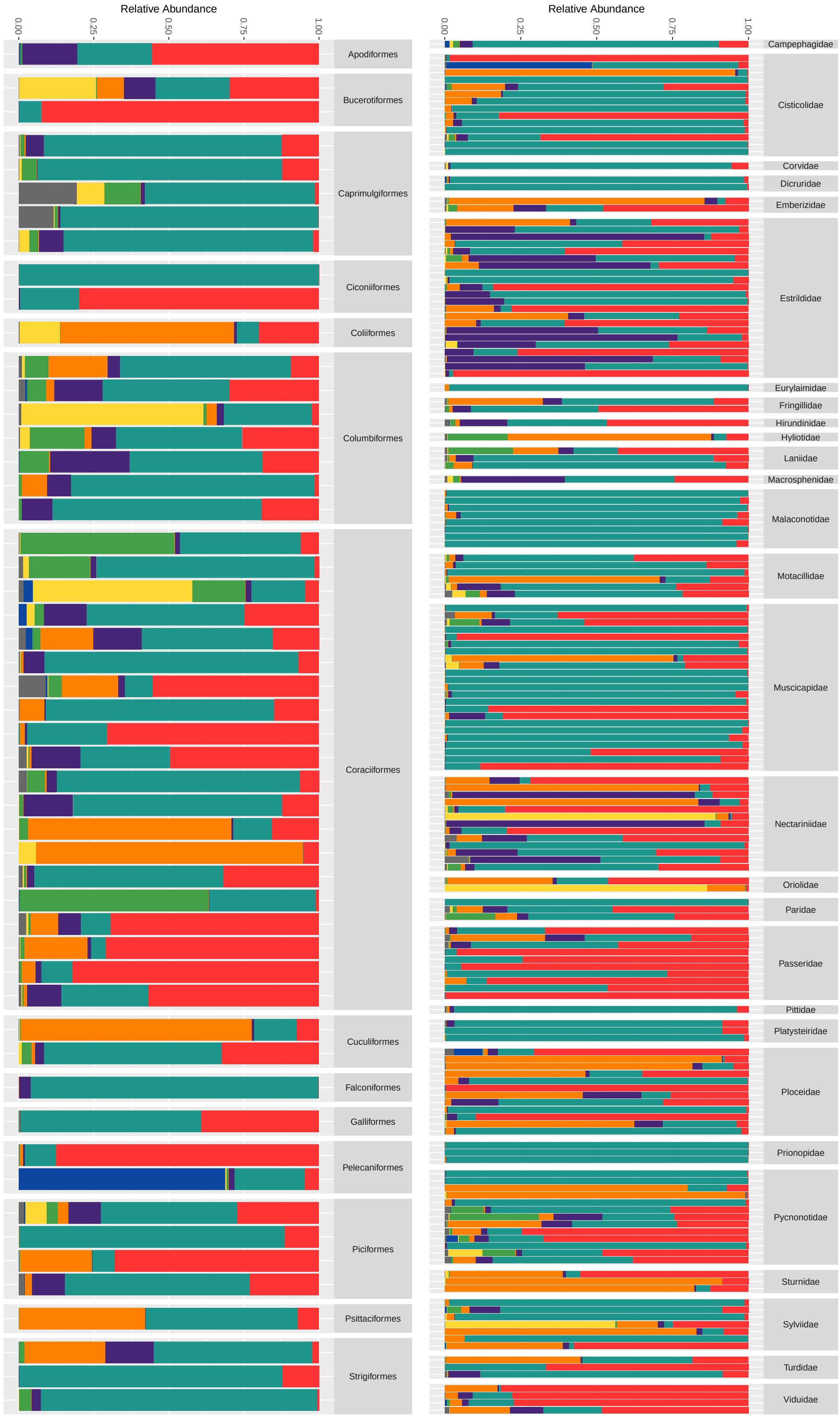

S2F Liver

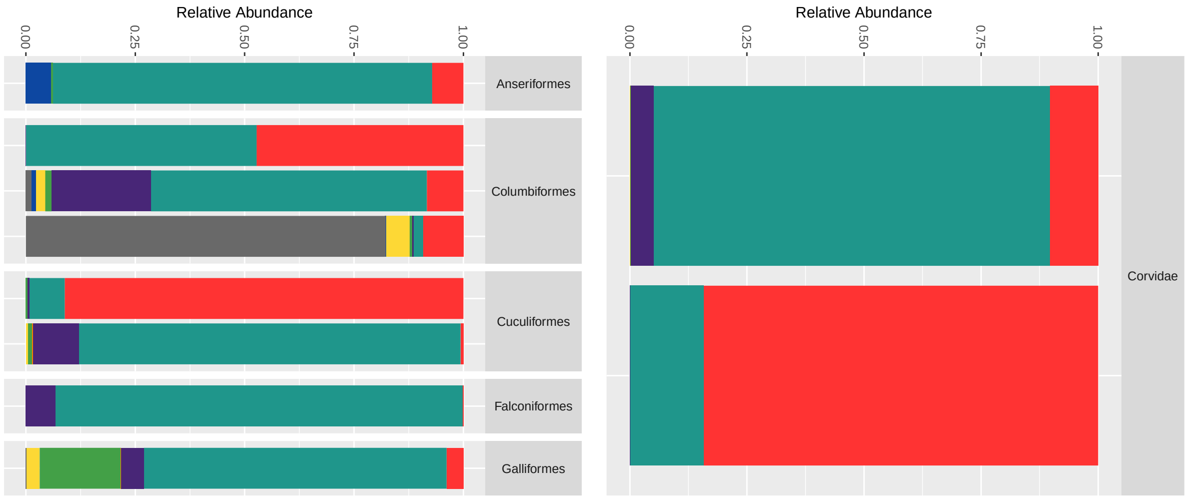

S2G Spleen

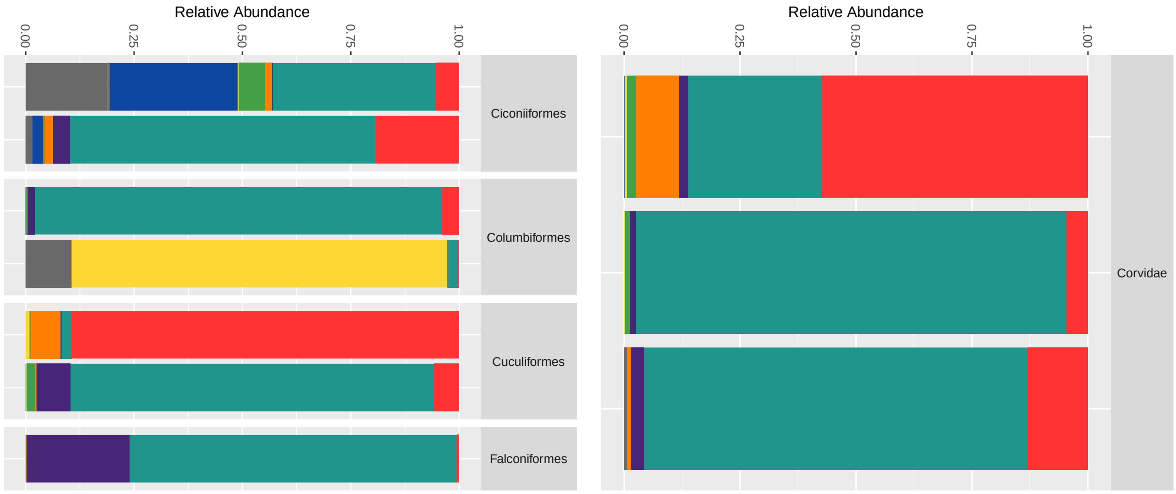

Figure S3. Alpha diversity calculations at the species level for species with more than three individuals, using Observed ASVs, Shannon and Simpson.

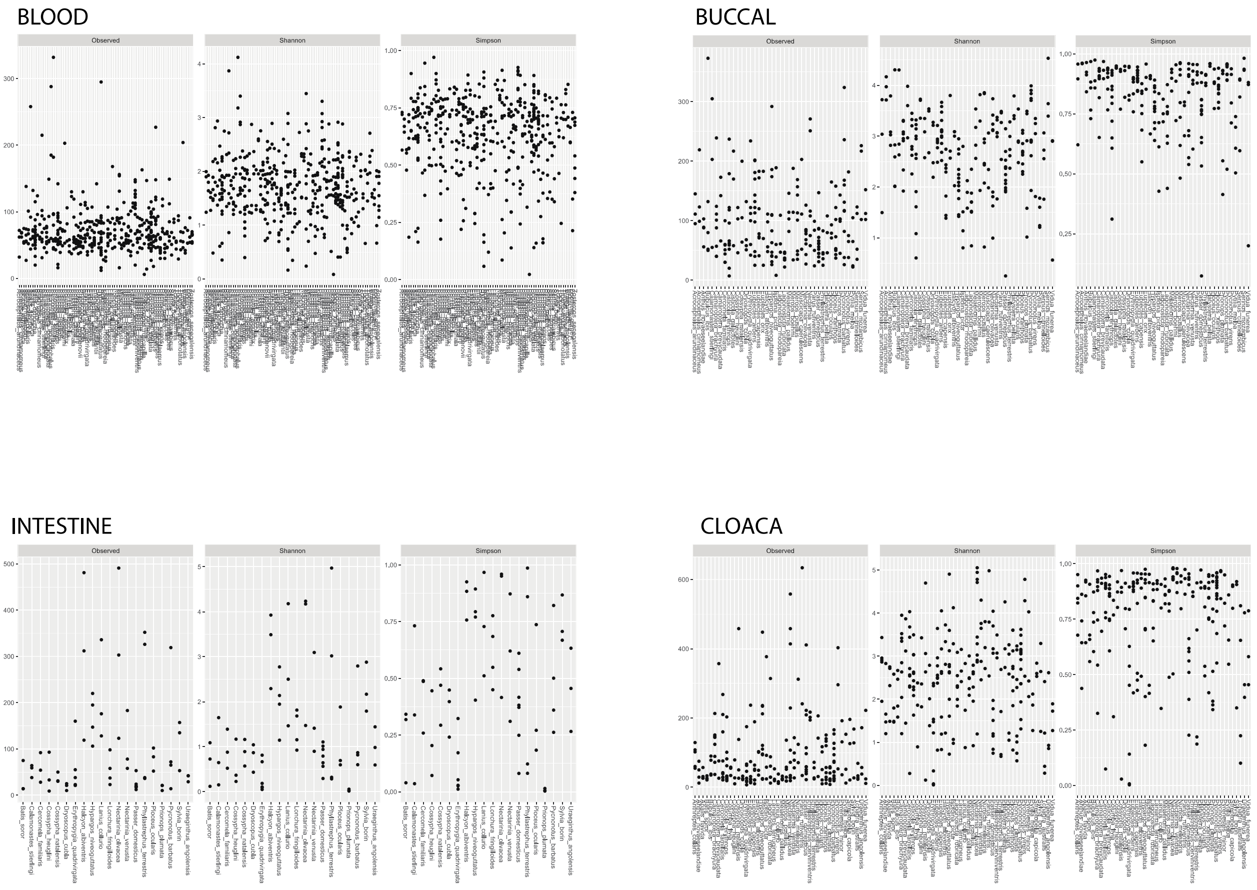

Figure S4. Alpha diversity plots for three body sites (blood, buccal, cloaca) for all species in the dataset with at least seven samples per body site.

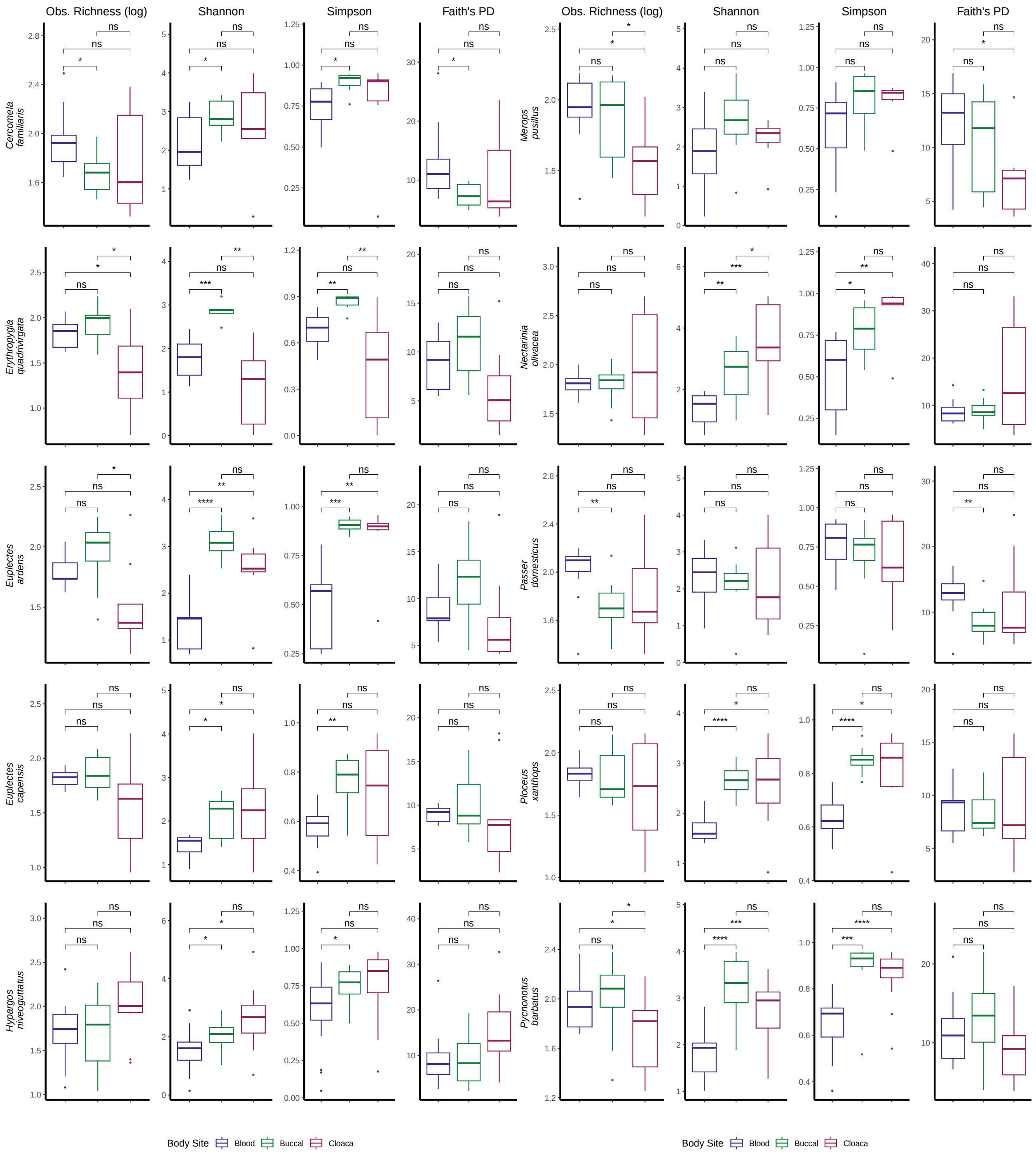

Figure S5. Comparison of cloacal and intestine samples after rarefaction to 9,000 sequences per sample. A. DESeq2 Plot showing differential abundance of ASVs, alpha=0.01, B. Venn diagram of shared and unique ASVs (left) and sequencing reads (right).

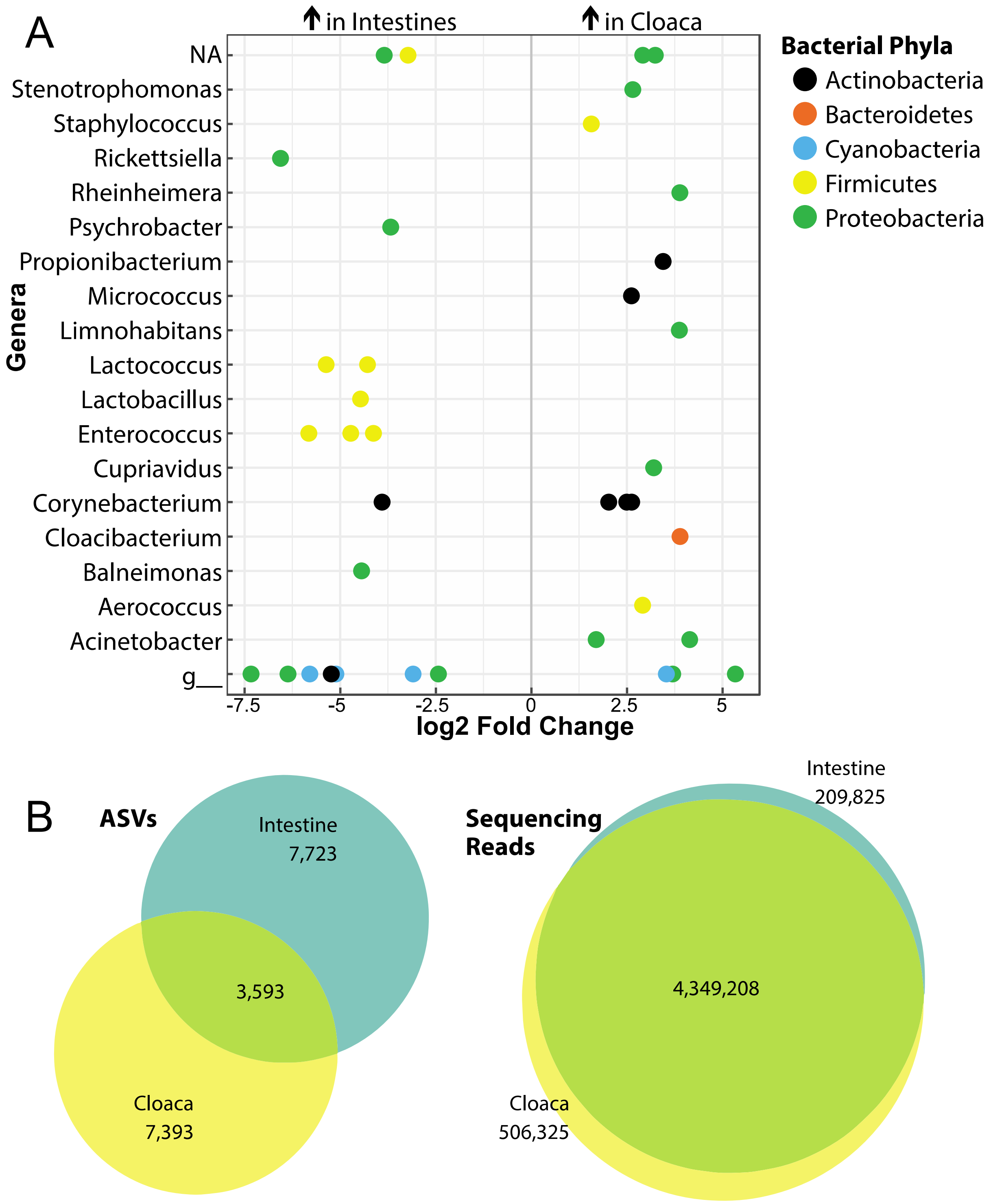

Figure S6. Tests for phylosymbiosis, using both weighted and unweighted UniFrac at the body sites contained in the "N>2" datasets, showed minimal congruence between host phylogeny and microbiota dendrograms. Pink bars indicate nodes shared by the two trees.

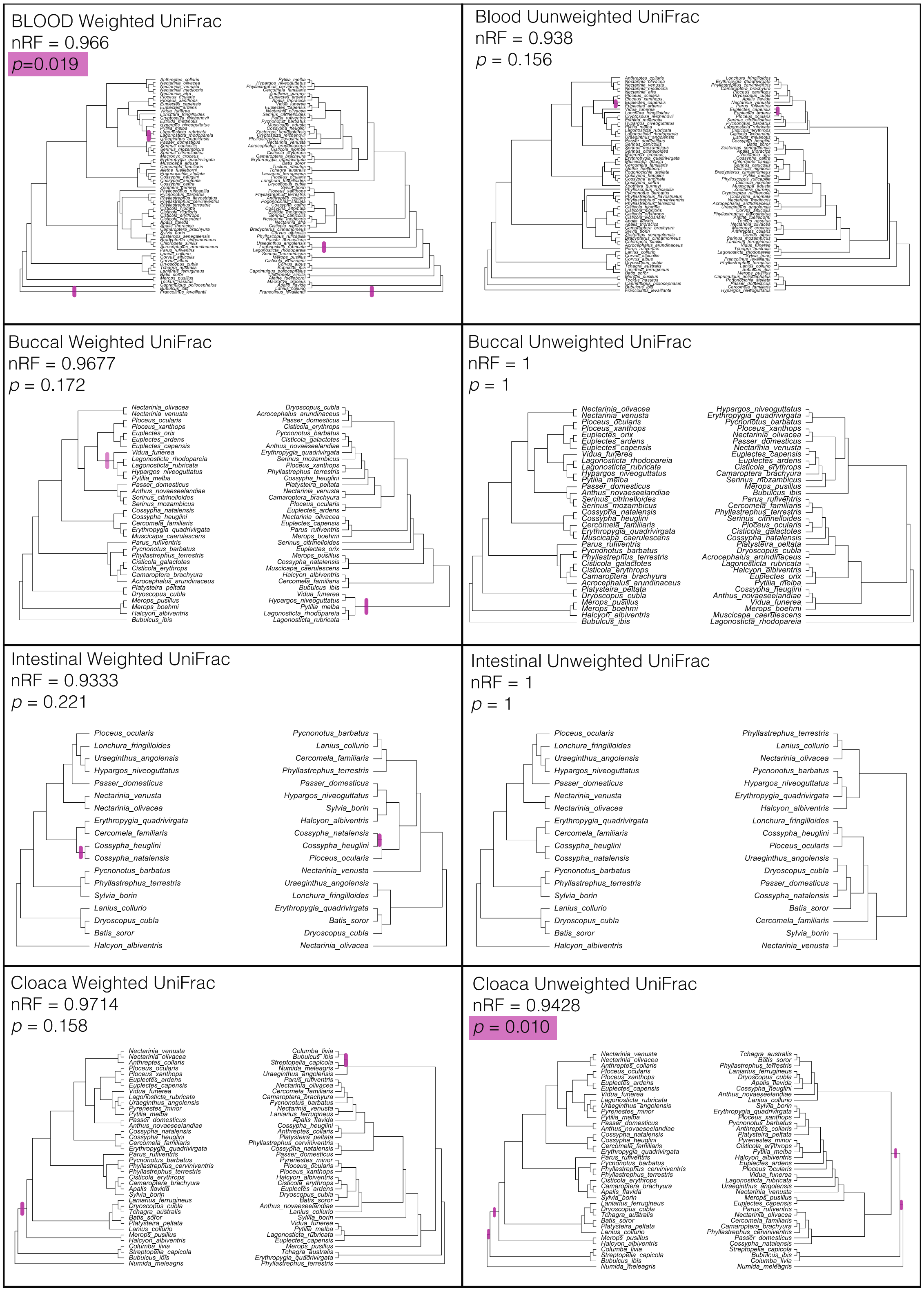

Supplement: Supplemental file 6 — Supplemental material. Download spectrum.03749-22-s0006.pdf, PDF file, 6.3 MB [file spectrum.03749-22-s0006.pdf]
